# Taxonomy of *Cyrtochilum*-alliance (Orchidaceae) in the light of molecular and morphological data

**DOI:** 10.1186/s40529-017-0164-z

**Published:** 2017-01-13

**Authors:** Dariusz L. Szlachetko, Marta Kolanowska, Aleksandra Naczk, Marcin Górniak, Magdalena Dudek, Piotr Rutkowski, Guy Chiron

**Affiliations:** 1 Department of Plant Taxonomy and Nature Conservation, The University of Gdańsk, ul. Wita Stwosza 59, 80-308 Gdańsk, Poland; 2 Department of Biodiversity Research, Global Change Research Institute AS CR, Bělidla 4a., 603 00 Brno, Czech Republic; 3 Department of Molecular Evolution, The University of Gdańsk, Wita Stwosza 59, 80-308 Gdańsk, Poland; 4 Herbiers, Université de Lyon I, 69622 Villeurbanne Cedex, France

**Keywords:** *Cyrtochilum*, Monophyly, New combinations, New species, Oncidiinae, Paraphyly, Taxonomy

## Abstract

**Background:**

The generic separateness and specific composition of the orchid genus *Cyrtochilum* was discussed for almost two centuries. Over the years several smaller taxa were segregated from this taxon, but their separateness was recently questioned based on molecular studies outcomes. The aim of our study was to revise concepts of morphological-based generic delimitation in *Cyrtochilum*-alliance and to compare it with the results of genetic analysis. We used phylogenetic framework in combination with phenetical analysis to provide proposal of the generic delimitation within *Cyrtochilum*-alliance. Two molecular markers, ITS and *mat*K were used to construct phylogenetic tree. A total of over 5000 herbarium specimens were included in the morphological examination and the phenetical analysis included 29 generative and vegetative characters.

**Results:**

Comparative morphology of the previously recognized genera: *Buesiella, Dasyglossum, Neodryas, Rusbyella, Siederella* and *Trigonochilum* is presented. A new species within the the latter genus is described. Fourteen new combinations are proposed. The key to the identification of the genera of the *Cyrtochilum*-alliance and morphological characteristics of each genus are provided.

**Conclusions:**

A total of six separated genera are recognized within *Cyrtochilum*-alliance. The reasons of the incompatibility between morphological differences observed within studied taxa and phylogenetic tree are argued and the taxonomic implications of such inconsistency, resulting in fragmentation or lumping of taxonomic units, are discussed.

**Electronic supplementary material:**

The online version of this article (doi:10.1186/s40529-017-0164-z) contains supplementary material, which is available to authorized users.

## Background

The genus *Cyrtochilum* was proposed in 1816 by German botanist C.S. Kunth along with descriptions of two new species, *Cyrtochilum flexuosum* Kunth and *Cyrtochilum undulatum* Kunth. Neither was designated as the generitype, which was standard procedure at that time. *C. undulatum* was selected as the type species of the genus by Garay (1974). Since its description, *Cyrtochilum* has been incorporated into the widely circumscribed genera *Oncidium* Sw. or *Odontoglossum* Kunth. by most subsequent taxonomists. The only exception was Kraenzlin (1917), who revitalized the genus a hundred years after its first description.

**Fig. 1:**
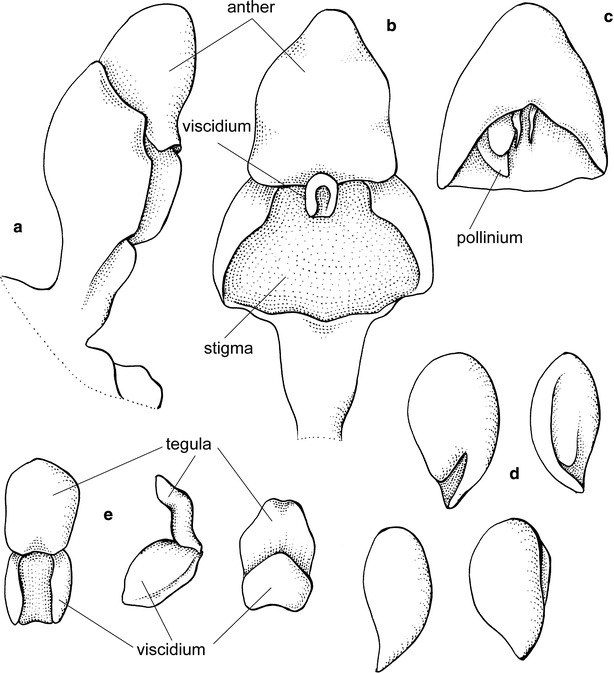
*Cyrtochilum volubile*. **a** Gynostemium, *side view*. **b** Gynostemium, *bottom view*. **c** Anther. **d** Pollinia, *various views*. **e** Tegula and viscidium, *various views* (Szlachetko & Mytnik-Ejsmont 2009)

*Cyrtochilum* once again became lost for over 80 years till Dalström (2001) reevaluated it and proposed several new nomenclatural combinations. The generitype determines somewhat the generic delimitation. According to this, *Cyrtochilum* should comprise species with flexuose, branching inflorescence, large flowers with broad, unguiculate sepals and petals, and narrow, slender lips covered in the basal part by large, massive, composed callus consisting of keels and digitate segments, and partially connate with a clavate, slender gynostemium, forming a right angle with the lip (Fig. 1).

On the basis of the sequences of molecular markers Neubig et al. (2012) proposed another circumscription of the genus. The authors included here various species, for example *Odontoglossum myanthum* Lindl. (generitype of *Dasyglossum* Königer & Schildh.), *Cyrtochilum flexuosum* Kunth (generitype of *Trigonochilum* Königer & Schildh.), *Oncidium aureum* Lindl. (generitype of *Siederella* Szlach., Mytnik, Górniak & Romowicz), as well as rspresentatives of *Rusbyella*, *Buesiella*, *Neodryas* and *Odontoglossum*. All of them inhabit mainly Ecuadorian Andes with many species also found in Colombian and northern Peruvian mountains. Neubig et al. (2012) created a monophyletic but highly heteromorphic unit, what resulted in the very enigmatic description of the genus (cf. Pridgeon et al. 2009; Dalström 2010).

The aim of presented study was to evaluate and compare morphological differences between taxa of *Cyrtochilum*-complex with the outcomes of molecular studies.

## Methods

### Morphological study

A total of over 5000 herbarium and liquid preserved specimens of orchids representing *Cyrtochilum s.l.* and related oncidioid genera and deposited in AMES, AMO, B, BM, C, COL, CUVC, F, FLAS, HUA, JAUM, K, MO, NY, P, PMA, UGDA, VALLE and W (Thiers 2015) were examined according to the standard procedures (database of specimens representing *Cyrtochilum s.l.* and *Odontoglossum* is provided in Additional file 1: Appendix S1). Every studied specimen was photographed and the data from the labels were taken. Both vegetative and generative characters of each plant were examined (the shape and size of the pseudobulbs, leaves, inflorescence architecture, shape and size of the floral bracts, flower morphology and gynostemium structure) and compared with existing type material of the most of distinguished species of the subtribe. The nomenclature of morphological characters follows Dressler (1981) and Szlachetko (1995).

### Phenetical analysis

Phenetical studies were employed based on 29 characteristics describing the taxonomically important generative and vegetative structures of *Cyrtochilum* species exploited by Neubig et al. (2012). As an outgroup we selected *Odontoglossum epidendroides*, a generitype of the genus *Odontoglossum*. A complete list of these features, as well as selected sets, is given in Additional file 2: Appendix S2. We have used a binary, 0–1, system of coding characteristics, because it is unambiguous and the most often applied in phenetic analyses. The incorporation of each feature for every *Cyrtochilum s.l.* species has resulted in a data matrix containing 1247 characteristics. To create hierarchic phenograms we used the PAST program (Hammer and Harper Ryan 2001). The so-called cluster analysis process is a typical method of analysis used in phenetic research (Stace 1989). We created a distance matrix using the Manhattan measure (Domański and Kęsy 2005; Pandit and Gupta 2011; Madhulatha 2012), which is an average subtraction measured across the dimensions ${\text{D}} =\Sigma {\text{i}}\left| {{\text{Xij}} - {\text{Xik}}} \right|$. We have also used the “middle links rule unweighted pair-group average” (UPGMA) as an amalgamation rule. The resulting phenograms were compared with the results of research conducted by Neubig et al. (2012).

### Molecular analyses

#### Taxon sampling

For the molecular analyses 91 specimens representing genus *Cyrtochilum.* The outgroup includes one species, *Odontoglossum epidendroides*. Sequences of outgroup taxa and for the most representatives of *Cyrtochilum* were downloaded from GenBank (Additional file 3: Appendix S3). DNA sequences of *Cyrtochilum volubile* were obtained in laboratory on the Department of Plant Taxonomy and Nature Conservation University of Gdansk. Sequences for both markers (ITS, *mat*K) were deposited in GenBank. Accession number and information about collector were place in Additional file 3: Appendix S3.

### Molecular markers

Nucleotide sequences from one nuclear (ITS) and one plastid (*mat*K) genome region were used in the molecular analyses. The ITS region consisted of the 18S and 26S ribosomal RNA genes, respectively the internal transcribed spacers (ITS1, ITS2) and the intervening gene 5.8S. For the sample of *Cyrtochilum volubile* was amplified part of the ITS region (ITS1 − 5.8S − ITS2) using the primers 101F and 102R (Douzery et al. 1999). For the *mat*K gene, we amplified fragment of approximately 1400 bp using the primers 19F (5′CGTTCTGACCATATTGCACTATG3′) from Molvary et al. (2000) and 1326R (5′TCTAGCACACGAAAGTCGAAGT3′) from Cuénoud et al. (2002).

### DNA extraction, amplification and sequencing

DNA was extracted using the Sherlock AX Kit (A&A Biotechnology, Poland) following manufacturer protocol. For the sample homogenization were used precooled in −45 °C lysing Matrix A tube and FastPrep instrument (MP Biomedicals, USA). Pellet of DNA was resuspended in 50 µl of TE buffer.

Amplifications and sequencing were using Eppendorf and Biometra TGradient thermal cyclers. PCR reaction for the both markers (ITS, *mat*K) were performed in a total volume of 25 µl containing 1 µl temple DNA (~10–100 ng), 0.5 µl of 10 µM of each primers, 12.0 µl Start Warm 2X PCR Master Mix (A&A Biotechnology, Poland), water and/or 1.0 µl dimethyl sulfoxide (DMSO) to ITS region/0.5 µl 25 mM MgCl2 only to *mat*K marker. Amplification parameters for nrITS (ITS1 + 5.8S + ITS2) were: 94 °C, 4 min; 30X (94 °C, 45 s; 52 °C, 45 s; 72 °C, 1 min); 72 °C, 7 min. For the part of *mat*K gene were: 95 °C, 3 min; 33X (94 °C, 45 s; 52 °C, 45 s, 72 °C, 2 min 30 s); 72 °C, 7 min. Wizaed SvGel and PCR Clean Up System (Promega, US) was used to clean PCR products following manufacturer protocol. Purified products of PCR reaction were cycle-sequenced using Big Dye Terminator v 3.1 Cycle Sequencing Kit (Applied Biosystems, Icn., ABI, Warrington, Cheshire, UK). Cycle sequencing parameters were: 95 °C, 2 min 40 s; 25X (95 °C, 10 s; 50 °C, 10 s; 60 °C, 4 min). Total volume sequencing reaction of 10 µl containing 1.3 µl of 5X sequencing buffer, 1 µl of Big Dye terminator, 0.4 µl of 10 µM primer (1.6/3.2 pmol), 0.5 µl dimethyl sulfoxide (DMSO), 1 µl of amplified product (30–90 ng/µl) and water. The sequencing reaction products were then purified and sequenced on an ABI 3720 automated capillary DNA sequencer in the Genomed S. A (Warsaw, Poland). DNA sequences chromatograms were inspected/edited in FintchTV and assembled using AutoAssembler (Applied Biosystems, Inc). Sequences for the *Cyrtochilum volubile* were deposited in GenBank (see Additional file 3: Appendix S3).

### Data analyses

The consensus sequences, both ITS region and part of *mat*K gene, were done automatically alignment by Seaview (Galtier et al. 1996) using algorithm MUSCLE (Edgar 2004). Analyses were performed separately on the matrix of each marker separately using PAUP*4.0b10 (Swofford 2002) and MrBayes 3.1.2 (Ronquist and Huelsenbeck 2003).

Maximum parsimony analysis (MP) used a heuristic search strategy with tree-bisection-reconnection (TBR) branch swapping and the MULTREES option in effect, simple addition and ACCTRAN optimization. Gaps were treated as a missing value. All characters were unordered and equally weighted (Fitch 1971). Internal support of clades was evaluated by character bootstrapping (Felsenstein 1985) using 1000 replicates. For bootstrap support levels, we considered bootstrap percentages (BP) of 50–70% as weak, 71–85% as moderate and >85% as strong (Kores et al. 2001). We also performed a Bayesian inference (BA). An evolutionary model for each region (ITS, *mat*K) was calculated with MrModeltest 2.2 (Nylander 2004). For the both data matrix the GTR + I + G model was selected according to the AIC (Akaike Information Criterion). For analyses, two simultaneous runs of four chains each were carried out with the MCMC algorithm, for 10,000,000 generations, sampling one tree for each 100, until the average standard deviation of split ranges was smaller than 0.01. After discarding the initial 25% trees of each chain as the burnin. Majority rule consensus tree was generation for the remaining trees in PAUP to assess topology and clades posterior probabilities (PP). Value of PP in Bayesian analysis are not equivalent to BP, generally are much higher (Erixon et al. 2003).

## Results

### Morphological analyses

**Fig. 2:**
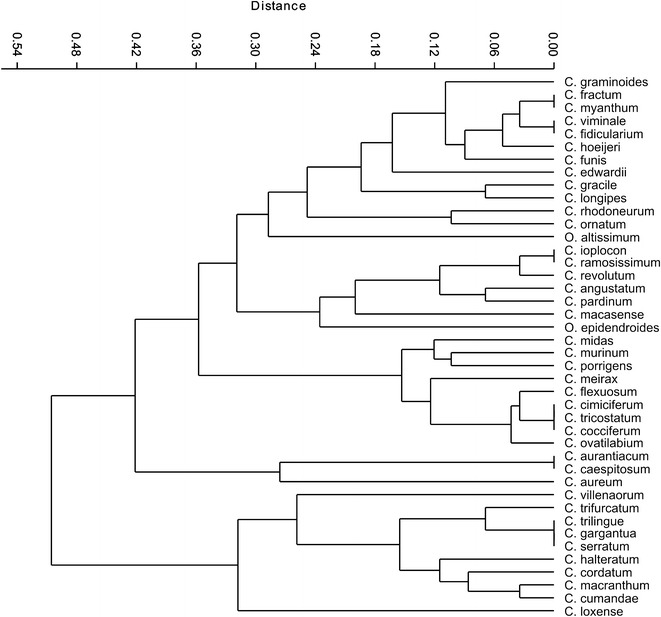
Phenetic similarity (UPGMA) of *Cyrtochilum s.l.* species based on morphological data

The phenetic similarity of the studied species based on morphological data is presented in Fig. 2. The first group comprises species usually classified to the genus *Dasyglossum* along with *Neodryas/Buesiella.* The species in this complex are characterized by subsimilar tepals, usually free sepals, an entire or 3-lobed lip, united basally with the base of the column, and parellal to it. The upper part of the lip is geniculate and often retrorse. The lip callus is simple, consisting of a pair of fleshy, parallel, adjoining tori, diverging in front, mostly enclosed by the thickened flanks of the gynostemium. The gynostemium is rather short, robust, in the upper half gently upcurved or straight. The generic borderline between *Dasyglossum* and *Neodryas/Buesiella* mostly concerns the character of the lip callus, which is large and variously lobed in the latter.

The next group includes *Cyrtochilum* species, such as “*C. ioplocon*”, “*C. ramosissimum*”, “*C. revolutum*”, “*C. angustatum*” and “*C. pardinum*”. All of these species are characterised by rather narrow, acuminate tepals with more or less undulate margins and somewhat twisted apices. Sepals and petals are dissimilar in form. Sepals have long and narrow claw, and petals—relatively short and wide. Lip is sessile, basally parallel to the gynostemium, and then geniculate bent down, the lamina is oblanceolate to oblong obovate in general outline, with acuminate and twisted apex. Lip calli consist of a pair of rather large basal wings with additional digitate or clavate projections below them. Gynostemium is erect, only basally connate with the lip, cylindrical, without any additional projections at the apex or at the base of the stigma. Floral bracts are usually shorter than half of pedicellate ovary. These species are mingled with *Odontoglossum epidendroides* and “*C. macasense*”. The former species is the type of the genus. Tepals of *Odontoglossum* are usually subsimilar, either set on prominent claw, or subsessile, but in both situations the claw of sepals and petals are similar. Margins of tepals are smooth, often crispate, and rarely undulate. Lip is basally connate with the gynostemium. In *O. epidendroides* the fusion is prominent and can reach one-fifth of the total lip length. Basal part of the lip is clawed, and lamina is more or less perpendicular to it. The shape of the lamina varies—usually it is oblanceolate to elliptic, often with crispate margins and long acuminate apex. Lip calli form a complicated pattern and consist of numerous digitate or lamellar projections, glabrous or ciliate. The gynostemium is usually somewhat arcuate, and form with the column an acute angle. It is apically adorned by various, filiform, digitate or lamellar projections. Floral bracts are prominently shorter than pedicellate ovary.

“*C. macasense*” is characterised by subsimilar, shortly clawed tepals, and sessile lip, which is prominently 3-lobed. The lip calli is compoused of two pairs of fleshy ridges of various lengths. The shorter pair is bilobed. Gynostemium forms an acute angle with the lip, and is erect, relatively short and massive, without any prominent appendages.

The “*C. midas*” group embraces species with small usually dull-coloured flowers, brownish or greenish-brown, which are usually treated as *Trigonochilum*. Tepals are rather dissimilar, sepals are narrower, with narrow claw, and petals are wider, short-clawed. The lip is triangular-cordate, sessile, diverging from the gynostemium at 70°–90° with a simple, torous, sometimes verrucose or gibbous callus. The lip lamina is centrally convex. The form and position of the gynostemium versus the lip in the species of this group is somewhat similar to *Cyrtochilum s.str*. It is usually elongate, basally much expanded and connate with the lip, slightly sigmoid or upcurved, slender, and the tegula has a prominent roof-like projection on the inner surface above the viscidium. We did not observe this character in any other species of the *Cyrtochilum*-clade. Floral bracts are rudimentary, much shorter than pedicellate ovary. *Trigonochilum* species are rarely confused with other genera, although the species boundaries are often not clear.

“*C. aurantiacum/caespitosum*” is rather an isolated group, at least as morphology is considered. Both are easily recognisable by the lip structure which has narrow, lower part, more or less canaliculated, with prominent rather simple calli. The apical part of the lip is much expanded forming transversely elliptic lamina. The gynostemium is somewhat similar to that one of *Dasyglossum*, i.e. it is erect, narrowly winged, apically upcurved. What is interesting tegula is narrow, linear 3–4 times longer than viscidium. Both species are included in the genus *Rusbyella.* Interestingly, “*C. aureum*” is linked to this group, although the gynostemium structure of “*C. aureum*” can suggest the affinity of this species to *Cyrtochilum s.str.* The short gynostemium is clavate, somewhat arcuate, with oblong-obovate projections with fringed margins. The gynostemium forms an acute angle with the lip. The lip reminds somewhat “*C. loxense*”, i.e. it is clawed, lamina is flat or convex, obscurely 3-lobed or pentagonal in outline, lip calli is missing to prominent, and contain of series of small projections in two rows. Lateral sepals are connate almost to the apex.

The last group contains those species which are included in the genus *Cyrtochilum s.str*. The common character of those species is gynostemium, gently sigmoid, basally prominently connate with the lip, elongated and slender above. The erect part is clavate and forms a right angle with the lip. The column part is slightly thickened just above the base, with two wing-like or digitate projections just below the stigma. Tepals are dissimilar, usually shield-like, obtuse to rounded apically, often undulate. Sepals have long and narrow claw, and petals—short and wide. At the base of the sepals’ claw wing-like appendices can be observed in most of the species. The lip of *Cyrtochilum s.str*. is sessile to shortly clawed, and usually divided into expanded and convex basal part and usually narrow, ligulate, pendent apical part. The lip calli is much complicated and usually consist of massive and variously lobed central part, with various number of additional projections spread all over the basal part. The floral bracts are large, leafy, nearly half as long as pedicellate ovary.

“*C. villenaorum*” is different from the species described above by the subsessile lip which has very large lamina, unequally 3-lobed, the middle lobe is more or less transversely elliptic in outline, with relatively small and simple calli with the middle lobe being somewhat upcurved. The gynostemium is devoid of any projections. Regarding morphology, “*C. volubile*” is very similar to “*C. villenaorum*”, but we did not include the former species in our analysis.

Morphologically distinct species in *Cyrtochilum s.str*. is “*C. loxense*”. Its tepals are subsimilar, shortly clawed; lateral sepals are connate in the basal fifth or so. Lip is straight, clawed, lamina is very unequally 3-lobed, with both lateral lobes relatively small, and the middle lobe very large, transversely elliptic with truncate apex. The calli is rather obscure and consist of series of irregular small projections near the lip base. The gynostemium is perpendicular to the lip, somewhat arcuate, basally connate with the lip claw, with short digital projections near the stigma.

### Molecular analyses

**Fig. 3:**
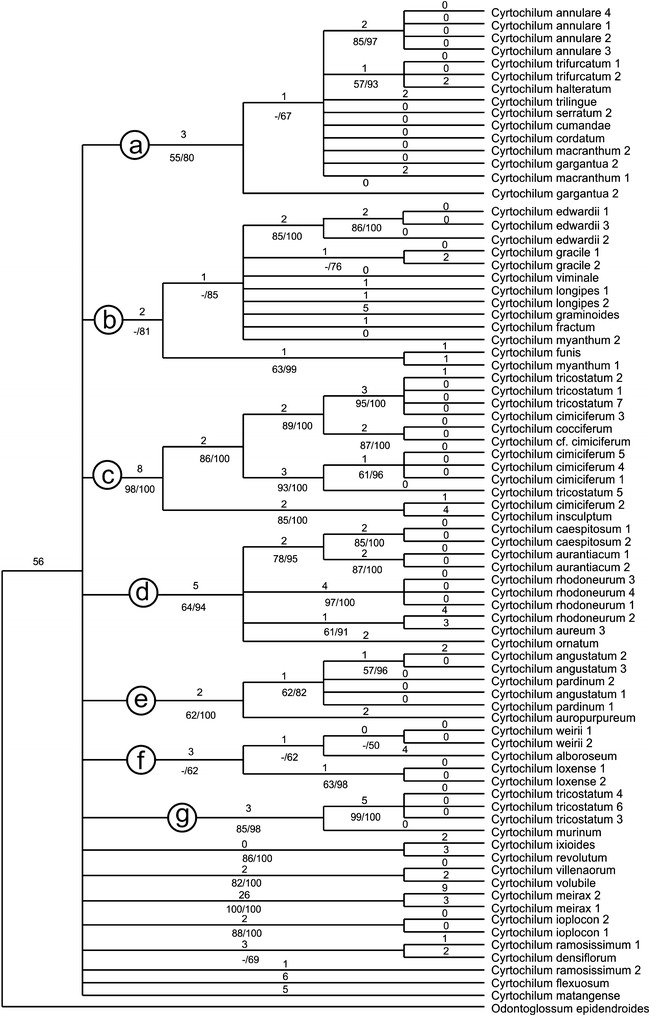
Bayesian 50% majority-rule tree for genus *Cyrtochilum* from ITS1-5.8S-ITS2 sequences. The *numbers* below the branches are bootstrap percentages (BP) and posterior probability (PP), bootstrap percentages ≥50% are given for supported clades. The branches length is shown above

**Fig. 4:**
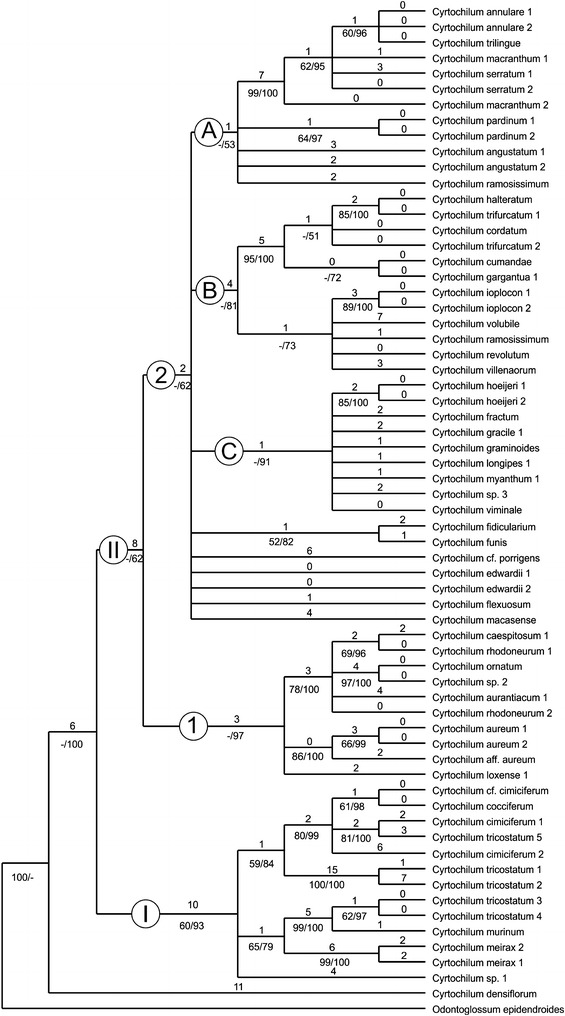
Majority-rule consensus of 7500 trees obtained in Bayesian analysis of *mat*K gene for genus *Cyrtochilum*. *Values* below branches represent bootstrap support (≥50%) from 1000 replicates and posteriori probabilities (≥50%) (BP/PP). The branches length is shown above

Statistcs for the data matrices (ITS, *mat*K) are separated by “/”. The number of analyzed taxa was 80/65 respectively. The aligned length of the matrix was 779/1303 characters of which 88/70 were parsimony informative. The number of the most parsimonious trees were >10.000, tree-length was 219/181, consistency index (CI) = 0.76/0.83 and retention index (RI) = 0.89/0.90. Consensus trees of Bayesian analysis are presented in Figs. 3 and 4.

Topology of MP trees and Bayesian trees are similar. The clades that have low bootstrap support or/and collapse in the strict consensus tree in parsimony analysis often appeared in Bayesian trees with low posterior probabilities too. One of the most parsimonious trees is available from the corresponding author. The combined phylogenetic tree presented by Neubig et al. (2012) is based on the analyses of five DNA regions (ITS, *trnH*-*psbA*, *5′ycf1*, *3′ycf1*, *matK*).

**Fig. 5:**
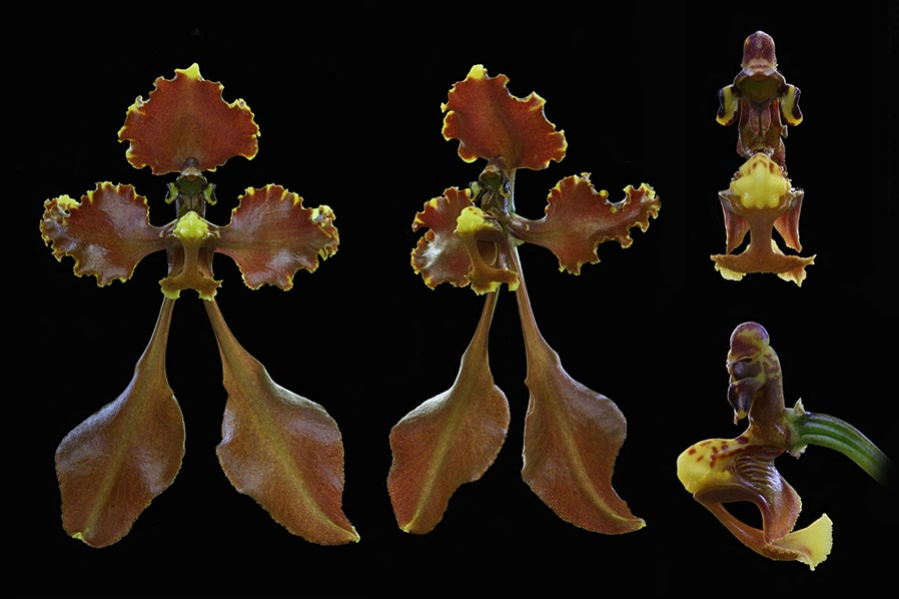
*Cyrtochilum cryptocopis*. Photo: Guido Deburghgraeve

**Fig. 6:**
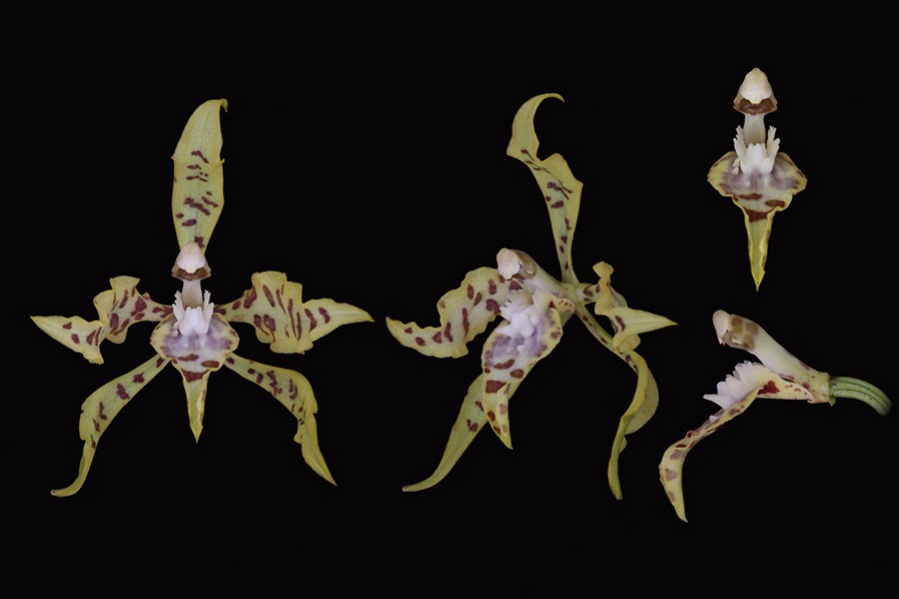
*Odontoglossum angustatum*. Photo by Guido Deburghgraeve

**Fig. 7:**
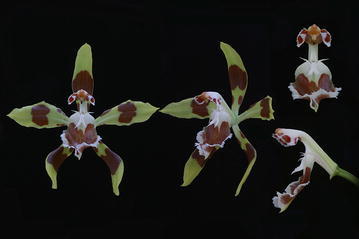
*Odontoglossum epidendroides*. Photo by Guido Deburghgraeve

**Fig. 8:**
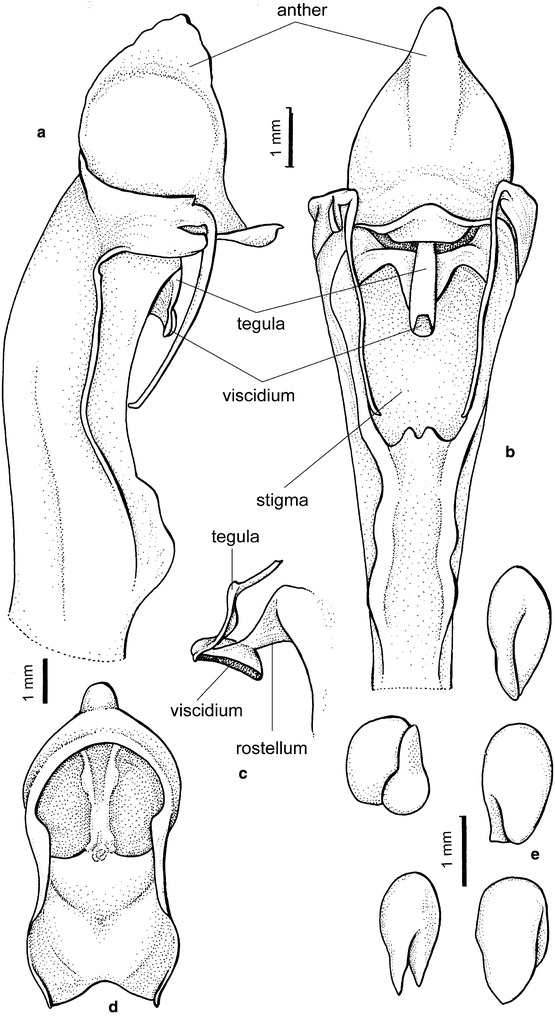
*Odontoglossum odoratum*. **a** Gynostemium, *side view*. **b** Gynostemium, *bottom view*. **c** Rostellum, *side view*. **d** Anther. **e** Pollinia, *various views* (Szlachetko & Mytnik-Ejsmont 2009).

The first subclade comprises the species of *Cyrtochilum s.str*. (Fig. 5) and “*C. ramosissimum*”, and is sister to the next subclade including two species—“*C. angustatum*” (Fig. 6) and “*C. pardinum*”. The last three aforementioned species resemble *Odontoglossum* typified by *Odontoglossum epidendroides* Kunth and, in fact, they have usually been assigned to that genus. It is noteworthy that *Odontoglossum epidendroides* is embedded in a separate clade (Fig. 7) and treated by Neubig et al. (2012) as a member of *Oncidium s.l*.. All the *Odontoglossum*-like species of *Cyrtochilum* mentioned above share a series of mutual features with *Odontoglossum*, i.a. gynostemium is slender, erect, forms an acute angle with a narrow lip, and it is fused with it along the midline at the base, creating two basal cavities (Fig. 8). The lip is geniculately bent near the middle exposing multiple calli consisting of narrow, digitate and/or filiform projections. Sepals and petals are narrow and undulate on margins, and sepals are prominently clawed.

In our *matK* tree species constituting this subclade form two groups A and B with posterior propability value 53 and 81, respectively. Group B comprises also *C. volubile* and *C. villenaorum*. The ITS tree does not solve relations between particular groups of species, although some branches are relatively highly or highly supported, e.g. *Cyrtochilum angustatum*–*C. pardinum* (e) with BS/PP = 62/100. Most other species of *Cyrtochilum s.str.* (a) are grouped together with BS/PP = 55/80.

**Fig. 9:**
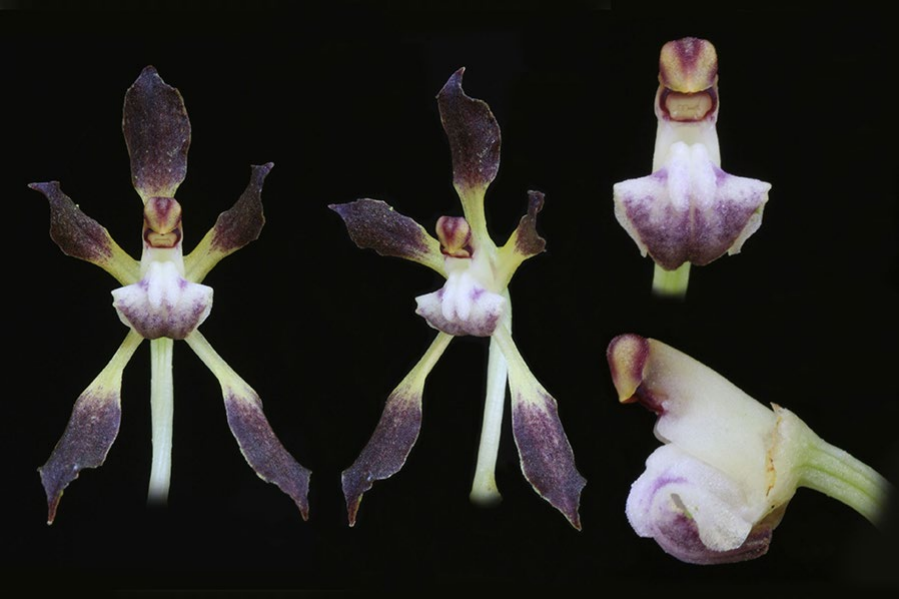
*Dasyglossum myanthum*. Photo: Guido Deburghgraeve

**Fig. 10:**
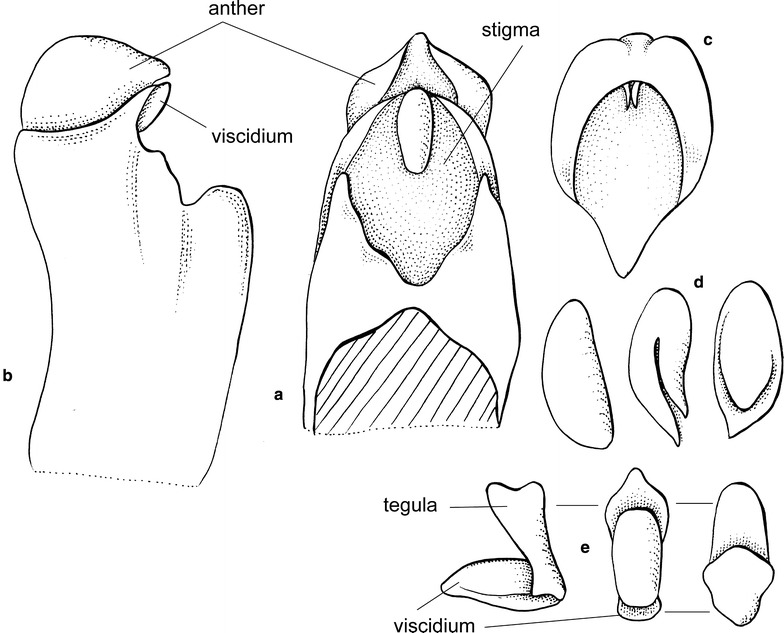
Dasyglossum myanthum. **a** Gynostemium, *bottom view*. **b** Gynostemium, *side view*. **c** Anther. **d** Pollinia, *various views*. **e** Tegula and viscidium, *various views* (Szlachetko & Mytnik-Ejsmont 2009)

The subclade *“Cyrtochilum myanthum*” includes species classified in *Dasyglossum* (Fig. 9), the genus established by Königer and Schildhauer (1994) and typified with *Odontoglossum myanthum* Lindl. The key characters of the genus mentioned by the authors are a simple callus, consisting of a pair of fleshy ridges and the lower half of the lip being parallel with the gynostemium, and apically part geniculately bent. Additionally, all species possess a massive, erect gynostemium, prominently winged and lateral sepals being free to the base (Fig. 10). The gynostemium and channeled lip callus form a kind of tube accessible to long-beaked pollinators.

The position of “*C. edwardii*” which is sister to *Dasyglossum* sublcade is unexpected, as it shares characters of the genus *Trigonochilum* rather than *Dasyglossum*, i.e. lip callus consisting of 7 massive projections confined to the central part of lamina, lip being arcuately bent down, and gynostemium and lip form a right angle. The colour of the flower, however, is unique for *Dasyglossum/Trigonochilum* alliance and is deep purple or lilac and lip callus is bright yellow. The gynostemium just below stigma is adorned with a pair of wing-like projections, not found in *Dasyglossum*.

It is interesting to note a position of “*C. flexuosum*”. The species is nested in two different places in cladogram; the first one is polytomic with *Dasyglossum* and “*C. edwardii*”, and the other one is embedded in *Trigonochilum* subclade. As the species is generitype of *Trigonochilum* we discuss it below.

“*C.* cf. *porrigens*” is again polytomic to the subclades mentioned above, and “*C. macasense*” is sister to all aforementioned groups. The first species is similar in all respects to *Trigonochilum* and has more or less triangular-obovate lip with complexed calli, clavate gynostemium basally connate with the lip and then abruptly upcurved in result forming an obtuse angle with it.The general flower architecture of *C. macasense*” reminds somewhat “*C. edwardii*”. The gynostemium and the lip form a right angle, lip callus consists of 4 ridges of various length, of which the shorter pair is bilobed. The colour of the flowers is a mixtre of yellow and brown, likes in *Trigonochilum*. The unique character of this species is prominently 3-lobed lip with much elongate middle lobe.

The *matK* tree does not solve relation between species of this subclade—some of them—e.g. *Cyrtochilum myanthum*, *C. viminale*, *C. gracile*, etc.—are grouped together (C) and highly supported (PP = 91). The others are polytomic, e.g. *C. edwardii*, *C. macasense* or *C. flexuosum*. All those species form a mutual subclade (b) in the ITS analysis (PP = 81).

**Fig. 11:**
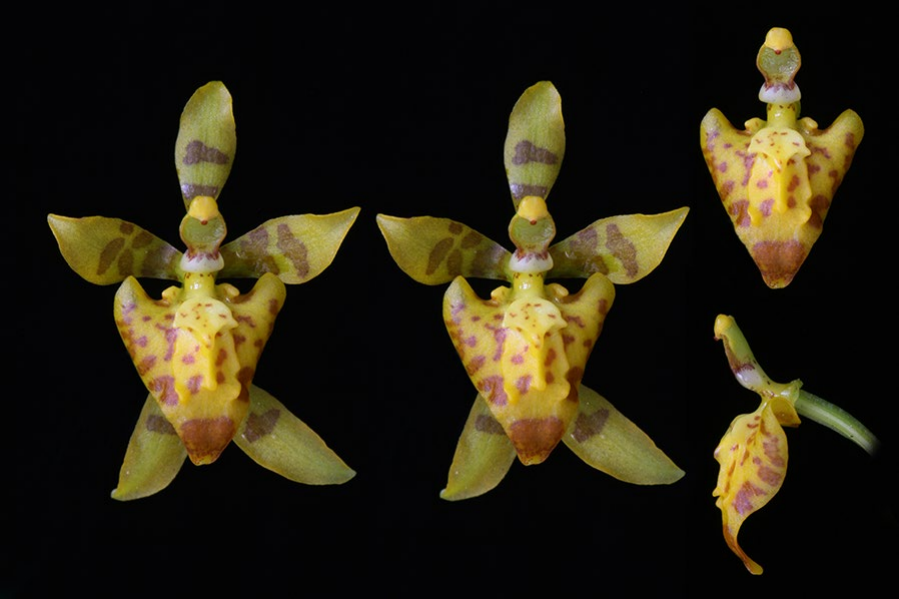
*Trigonochilum meirax*. Photo: Guido Deburghgraeve

**Fig. 12:**
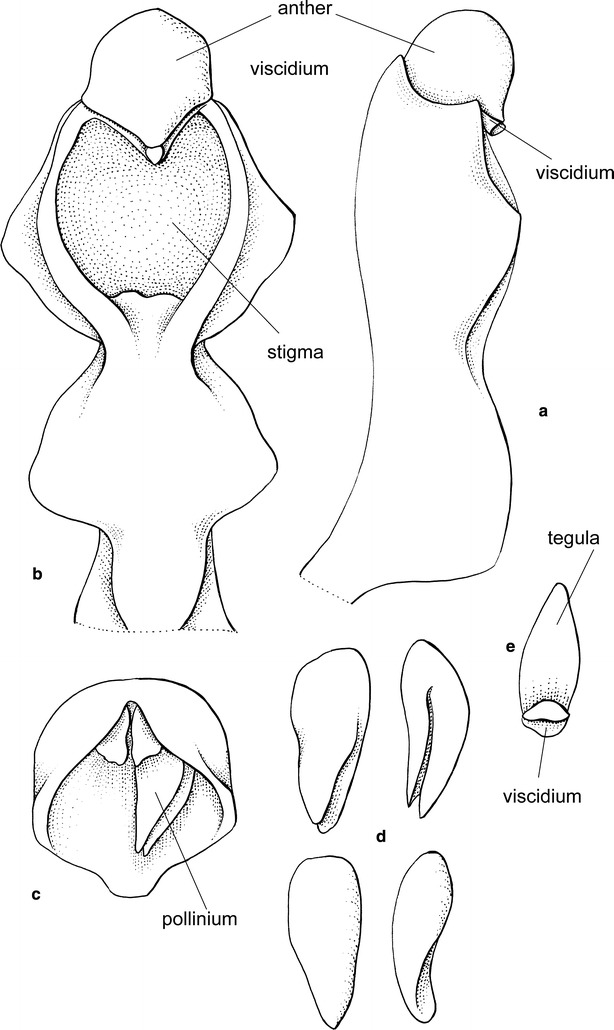
Trigonochilum meirax. **a** Gynostemium, *bottom view*. **b** Gynostemium, *side view*. **c** Anther. **d** Pollinia, *various views*. **e** Tegula and viscidium (Szlachetko & Mytnik-Ejsmont 2009)

The subclade “*Cyrtochilum flexuosum*” embraces species assigned to the genus *Trigonochilum* (Fig. 11). The genus was described in 1994 by Königer and Schildhauer to encompass Oncidiinae species characterized by a subtriangular lip diverging from the gynostemium at 70°–90° and a short, stout, clavate gynostemium (Fig. 12) with distinct swellings below the stigma. The lip callus is a large mass of variously, but shallowly lobed tissue occupying the central part of the lamina. The authors designated *T*. *flexuosum* (Kunth) Königer & Schildh. as a generitype and presented a list of 22 species transferred to the newly established taxon from *Cyrtochilum* Kunth, *Odontoglossum* Kunth and *Oncidium* Sw. In the following years, Königer (1996, 1999, 2000) described some new species of *Trigonochilum* and other species were reassigned to the genus or described by Senghas (2001, 2003). The latter author, however, synonymized all the species of *Dasyglossum* Königer under *Trigonochilum*. With some additional transfers made by Königer (2008, 2010) and a description of the new species, the genus currently includes about 60 species with a distribution from Peru and Bolivia to Colombia and Venezuela. The border between them is very often very difficult to define. Dualistic position of “*C. flexuosum*” on the Neubig et al. (2012) phylogenetic tree is probably caused by misidentification of one of the samples.

The species constituting this subclade are on the mutual branch (I) in *matK* tree and has 60/93 BS/PP. This branch is sister to all other *Cyrtochilum*-alliances. The ITS tree analysis gives somewhat different pattern of relation between aforementioned species—this subclade is divided into two groups c and g, with high bootstrap support and posterior propability—98/100 and 85/98, respectively. Relations between these groups are not solved.

**Fig. 13:**
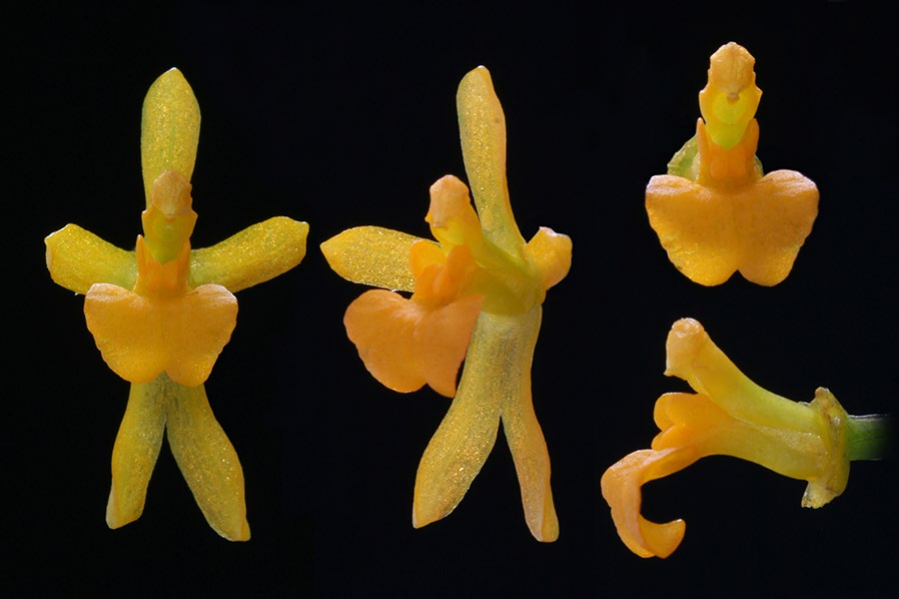
*Rusbyella aurantiaca*. Photo: Guido Deburghgraeve

**Fig. 14:**
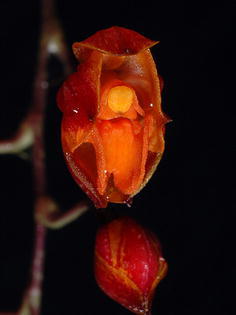
*Neodryas rhodoneura*. Photo: Eric Hunt

**Fig. 15:**
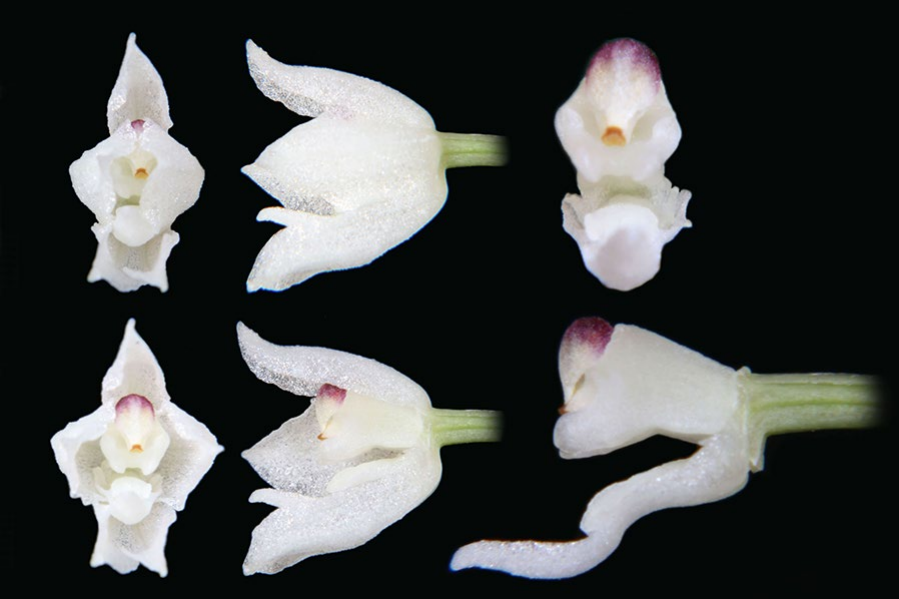
*Neodryas schildhaueri*. Photo: Guido Deburghgraeve

**Fig. 16:**
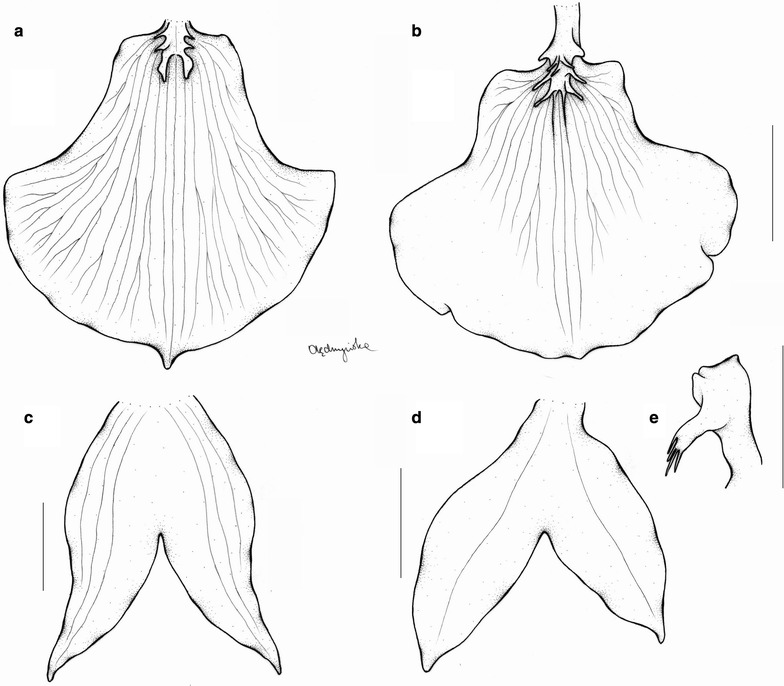
*Siederella aurea.* **a**, **b** Lip, **c**, **d** lateral sepals, **e** gynostemium. Drawn by N. Olędrzyńska. *Scale bar* 5 mm

**Fig. 17:**
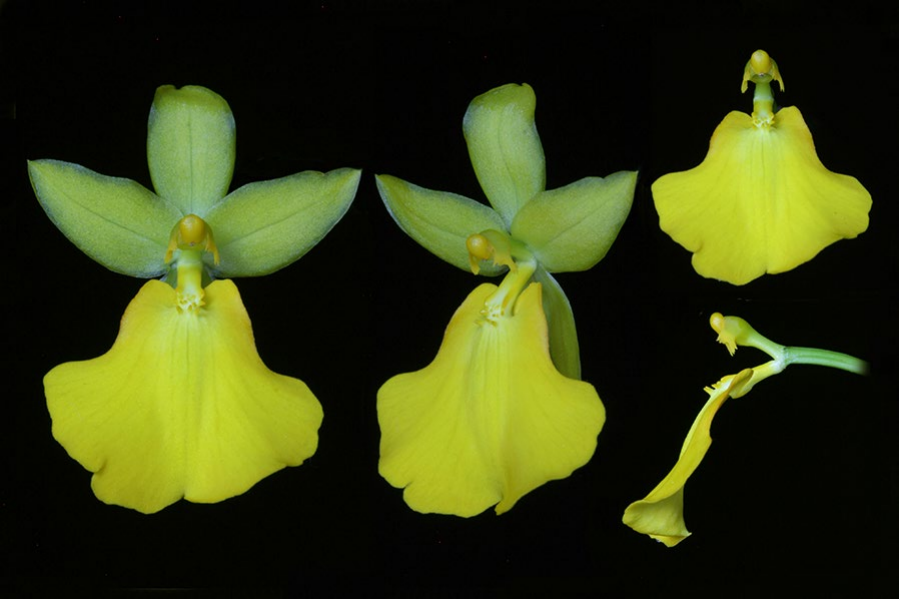
*Siederella aurea.* Photo: Guido Deburghgraeve

**Fig. 18:**
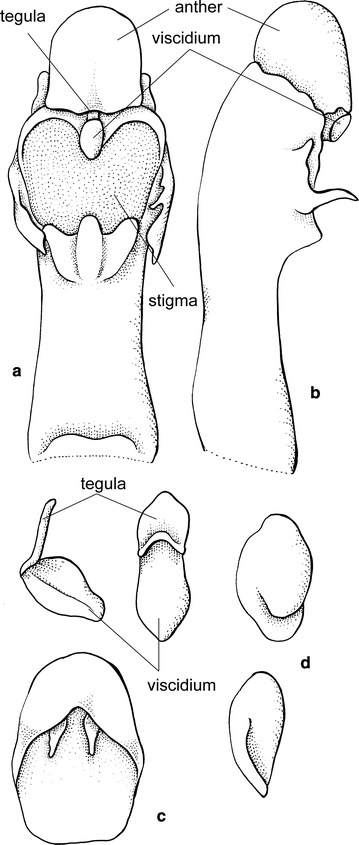
Siederella aurea. **a** Gynostemium, *bottom view*. **b** Gynostemium, *side view*. **c** Anther. **d** Pollinia, *various views* (Szlachetko & Mytnik-Ejsmont 2009)

The last subclade of the *Cyrtochilum*-group is composed of a mixture of species included in various genera, whose common features are more or less connate lateral sepals, stout gynostemium, usually parallel to the lower part of the lip, and bent in the geniculate manner above base. “*C. aurantiacum*” and “*C. caespitosum*” are easily distinguishable from all other *Cyrtochilum* species by their lip structure, i.e. a narrow, canaliculated claw occupied by an oblong callus, expanded apically in transversely elliptic lamina. The gynostemium is straight and apically reflexed. These species have been classified in the genus *Rusbyella* (Fig. 13). “*C. rhodoneurum*” differs from the aforementioned species in its oblong-ligulate lip with a prominent central callus. It has been assigned to the genus *Neodryas* (Figs. 14, 15). “*C. ornatum*”, usually included in the genus *Buesiella*, are distinguished from the above species by their digitate projections near the receptive surface and a hastate lip. “*C. aureum*” was the only species of the genus *Siederella* characterized by a narrowly clawed lip with greatly expanded lamina (Fig. 16), a rather obscure central callus and digitate projections near the stigma (Fig. 17). The gynostemium forms an angle of ca 30° with the lip (Fig. 18). In both analysed trees based on ITS (d) and *matK* (1) aforementioned species are grouped together with high PP value—94 and 97, respectively. In this case bootstrap suppor is low (64 and <50).

**Fig. 19:**
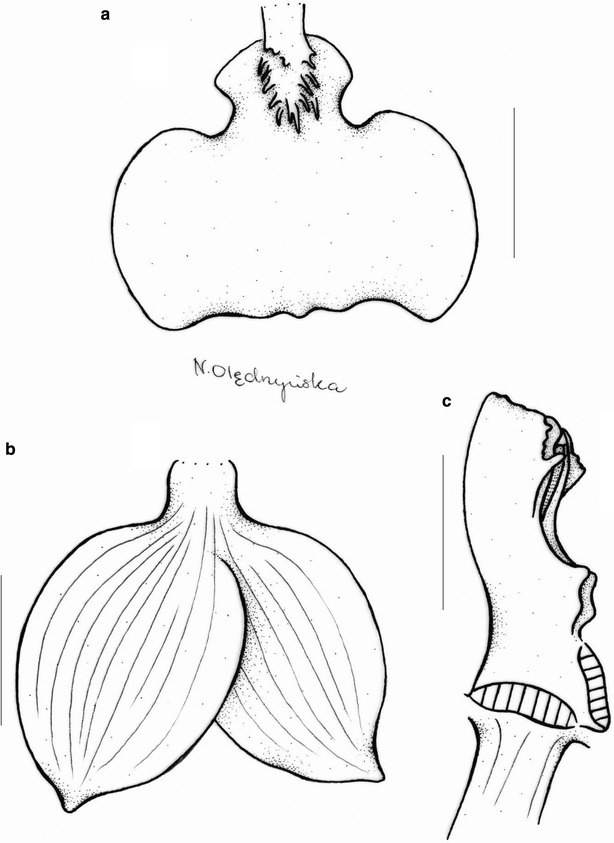
*Oncidium loxense.* **a** Lip, **b** lateral sepals, **c** lip. Drawn by N. Olędrzyńska. *Scale bar* **a**, **b** = 10 mm, **c** = 5 mm

**Fig. 20:**
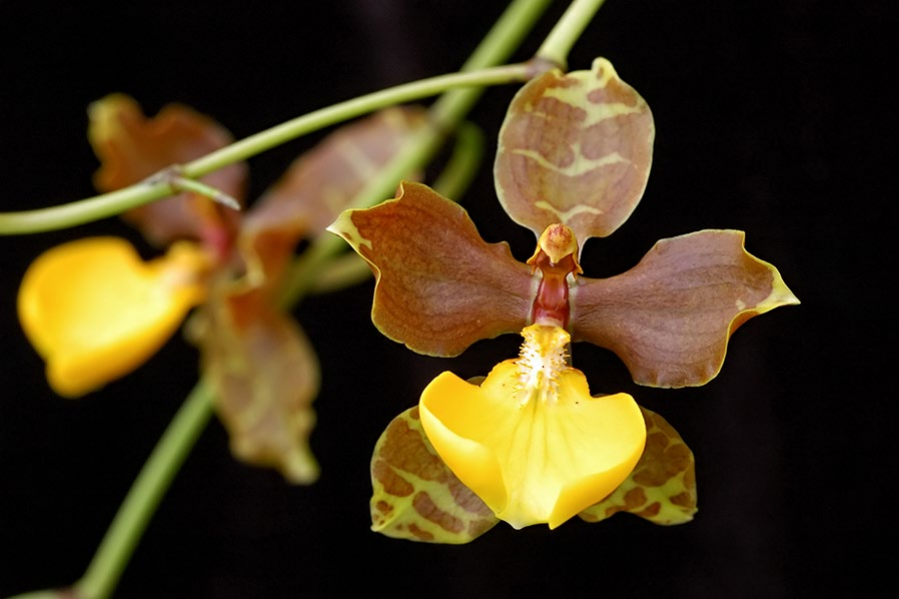
*Oncidium loxense.* Photo: Eric Hunt

The last species in the group is “*C. loxense*” (Figs. 19, 20), which in habit, type of inflorescence and clawed tepals is reminiscent of *Cyrtochilum s.str*. Even though its gynostemium is perpendicular to the lip, the labellum is unique in the genus—it is short-clawed, 3-lobed with the middle lobe being the largest, transversely elliptic and concave. The lip callus is relatively small and confined to the basal part of the lip. In *matK* tree *C. loxense* is attached to *C. caespitosum*-alliance (1), and in the ITS tree this species is connected with *C. alboroseum* and *C. weirii* (f). In the first case value of posterior propabilty is high (97) and in the second—only 62. The *matK* shows that *C. loxense* is only distantly related with *C. villeanorum* and *C. volubile*, with which it is very similar morphologically. The relations between these species are not solved in out ITS analysis.

## Discussion

Until recently, it appeared that DNA fragment sequencing would enable the reconstruction of the phylogeny of organisms with a high degree of accuracy. Almost all data obtained from any sources other than genetic material began to be discarded. Numerous articles presenting a completely new approach to the taxonomy of plants and other organisms were published (e.g. Chase et al. 2000; Asmussen et al. 2006; Friesen et al. 2006; Lefébure et al. 2006). In many cases, the new classifications overturned those proposed earlier. Interestingly, one can note a disagreement between molecular based systems and morphological ones. Usually, priority was given to those based on the results of DNA fragment analyses, even though relatively often it was difficult or even impossible to interpret the topology of the tree in terms of its morphology. Yet, no systems based on limited datasets reflect the evolution of the whole organisms; rather, they focus just on the evolutionary modifications of the data in question. Using phylogenetic data to study speciation requires that potential limitations be kept in mind. The approach assumes that we have an accurate and complete understanding of the evolutionary relationships within a clade. Solid phylogenetic methods and markers are needed to reconstruct the phylogeny, which is often difficult, especially among recently diverged taxa.

The utility of nuclear gene sequences in intraspecific phylogenetic analyses appears to be limited by increased coalescence time as compared to chloroplast genes. In addition, the potential for reticulate evolution among nuclear alleles due to recombination is likely to further limit their utility for phylogenetic studies (Bermingham and Moritz 1998). When using organellar genes in combination with nuclear genes, several factors contribute towards an increase in the genetic structure encountered within plant species. For phylogenetic purposes, it would be desirable to consider multiple gene trees based on chloroplast and nuclear genomes, because independently derived gene trees may not be congruent (Schaal et al. 1998). However, Doyle (1997) notes that when the history of the organellar genome is different from that of the nuclear genome (e.g. in lineage sorting or introgression) every comparison sequence in these genomes will give a false phylogenetic pattern for those taxa, and this can confound phylogenetic reconstruction. Plant molecular phylogenetic studies at species levels are generally limited by the availability of sequences with levels of resolution suitable for the construction of well-supported trees (Doyle et al. 1996).

Defining *Cyrtochilum s.l.* Neubig et al. (2012) stated that “vegetatively *Cyrtochilum* are distinguished by dull pseudobulbs that are round or ovoid in cross section with two to four apical leaves and two to six leaf-bearing sheaths and relatively thick roots, in contrast *Oncidium* spp. have glossy, ancipitous (two-edged) pseudobulbs and thin roots”. Unfortunately, characters mentioned by Neubig et al. (2012) do not warrant proper identification of *Cyrtochilum*, since the features selected by the authors as disciminative can be found also in other Oncidiinae, for example in *Brassia s.l*.

A problem has emerged as to how to explain the similarity between molecular marker sequences in morphologically different species, such as *Cyrtochilum s.str*. and “*Cyrtochilum ramosissimum”* or “*C. angustatum”*, which together form a common phylogenetic branch. Neubig et al. (2012) stated that great variability in the flower architecture in Oncidiinae probably reflect a shift in pollinators. On the other hand, morphological similarity between phylogenetically distantly related taxa can be explained by homoplasy. It cannot be excluded, however, that the explanation is much more complicated.

There are at least some phenomena which can usher generate a disturbance to the topology of the phylogenetic tree. Ancestral hybridization, polyploidization and hybrid speciation are significant evolutionary forces in the Orchidaceae. Numerous examples of hybrids are noted in this group of plants. Interspecific hybrids occur in Orchidaceae, but they are typically sporadic and local (e.g. Cozzolino and Aceto 1994; Cozzolino et al. 1998). On the other hand, some putative orchid hybrids are more widespread and stabilized (e.g. Hedrén 1996, 2001; Arft and Ranker 1998; Bullini et al. 2001). Most polyploid species have formed recurrently from genetically-distinct diploid progenitors, representing a potentially great gene pool for the derivative polyploid. Relatively recent hybrid-derived species disclose some degree of morphological intermediacy between putative parents or a similarity to one of the parents. Furthermore, such deviation from intermediacy may be expected in a stablilized hybrid that has been under various selective pressures (Goldman et al. 2004).

A genomic investigation has demonstrated that polyploidization and hybridization are highly effective evolutionary mechanisms for introducing new plant species, promoting their persistence, and ultimately increasing the diversity of plant species (Cook et al. 1998; Ramsey and Schemske 1998; Soltis and Soltis 1999; Otto and Witton 2000; Wendel 2000; Hewitt 2001). While hybridization can be a threat to species integrity, it can also be a source of new variation and a source of new species, especially through polyploidy (Grant 1981).

The stability of the polyploid genome depends on non-random genetic changes, including chromosome and genome gains and losses of loci. This genomic reorganization seems to proceed quickly (Rieseberg et al. 1996; Rieseberg 1997; Buerkle and Rieseberg 2008), for example, after 10–60 generations in the case of *Helianthus anomalus* (Ungerer et al. 1998).

Hybrid speciation appears to be facilitated by several additional factors, for example, availability of a suitable ecological niche or development of appropriate fitness (Rieseberg 1997; Mallet 2007). To be evolutionarily successful, even fertile and stable hybrids must be reproductively isolated from the parental species either by chromosomal sterility factors, or evolution of reproductive barriers, or divergence into a new ecological niche (Grant 1981; Rieseberg 1997, 2001; Wu 2001; Paun et al. 2009).

In the case of species of hybrid origin, we expect the conflict of the topology between nuclear and plastid genes. Below we explain the mechanisms leading to these conflicts. Consider a situation of conflict between molecular and morphological data. There are two possibilities of such cases. The first concerns the situation where two related taxa differ morphologically due to the divergent evolution resulting as adaptation to different habitats and/or pollinators. The second case explains this phenomenon by referring to the convergence that results from adaptation to a common pollinators. But there is a third solution to the conflict—reticulate evolution. To make its detection should be compared to a tree topologies based on plastid and nuclear sequences. The phylogenetic analyses conducted by Neubig et al. (2012) used a nuclear marker: ITS1-5.8S-ITS2, which is part of a family of genes coding for ribosomal DNA (rDNA). In higher plant rDNA is organized into arrays at one or more chromosomal locations (Rogers and Bendich 1987; Hillis and Dixon 1991). Each array contains hundreds to thousands of identical to near-identical repeats. The repeats having become homogenized by evolutionary forces like unequal crossing-over (Seperack et al. 1988) or gene conversion (Enea and Corredor 1991; Hillis et al. 1991) that are referred to as concerted evolution (Zimmer et al. 1980; Arnheim et al. 1980). Wendel et al. (1995) observed complete or nearly complete interlocus concerted evolution of the ITS region in diploid and polyploidy *Gossypium* species. Moreover, they observed this phenomenon in other components of rDNA repeat. These authors also observed, that concerted evolution has occurred bidirectionally in analysed species. One of the five polyploid hybrids (*Gossypium mustelinum*) had rDNA repeat from female parent (receiver of the pollen) and remaining polyploids (*G*. *tomentosum*, *G*. *hirsutum*, *G*. *darwini*, *G*. *raimondii*) has become homogenized to a male parent (donor of the pollen) rDNA repeat. In such case, ITS sequences from the allopolyploid species occur on both branches of phylogenetic tree, each close to the one of the parental species. In case of maternally inherited plastid DNA, which occurs in most angiosperms, both rDNA lineage has the same plastid DNA lineage. So we can detect species of hybrid origin only with one of this case. The use of nuclear ITS sequences in phylogenetic analyses for the species of hybrid origin may lead to an underestimation of the phylogeny. Due to the frequent occurrence of hybridization in plants, especially in orchids seems to be a reasonable use of the other nuclear markers in order to properly assess the phylogenetic relationship between the analysed taxa. Low-copy nuclear genes, which are less liable to concerted evolution, can potentially serve as a very useful marker for reconstructing allopolyploidization (Small et al. 1998).

**Fig. 21:**
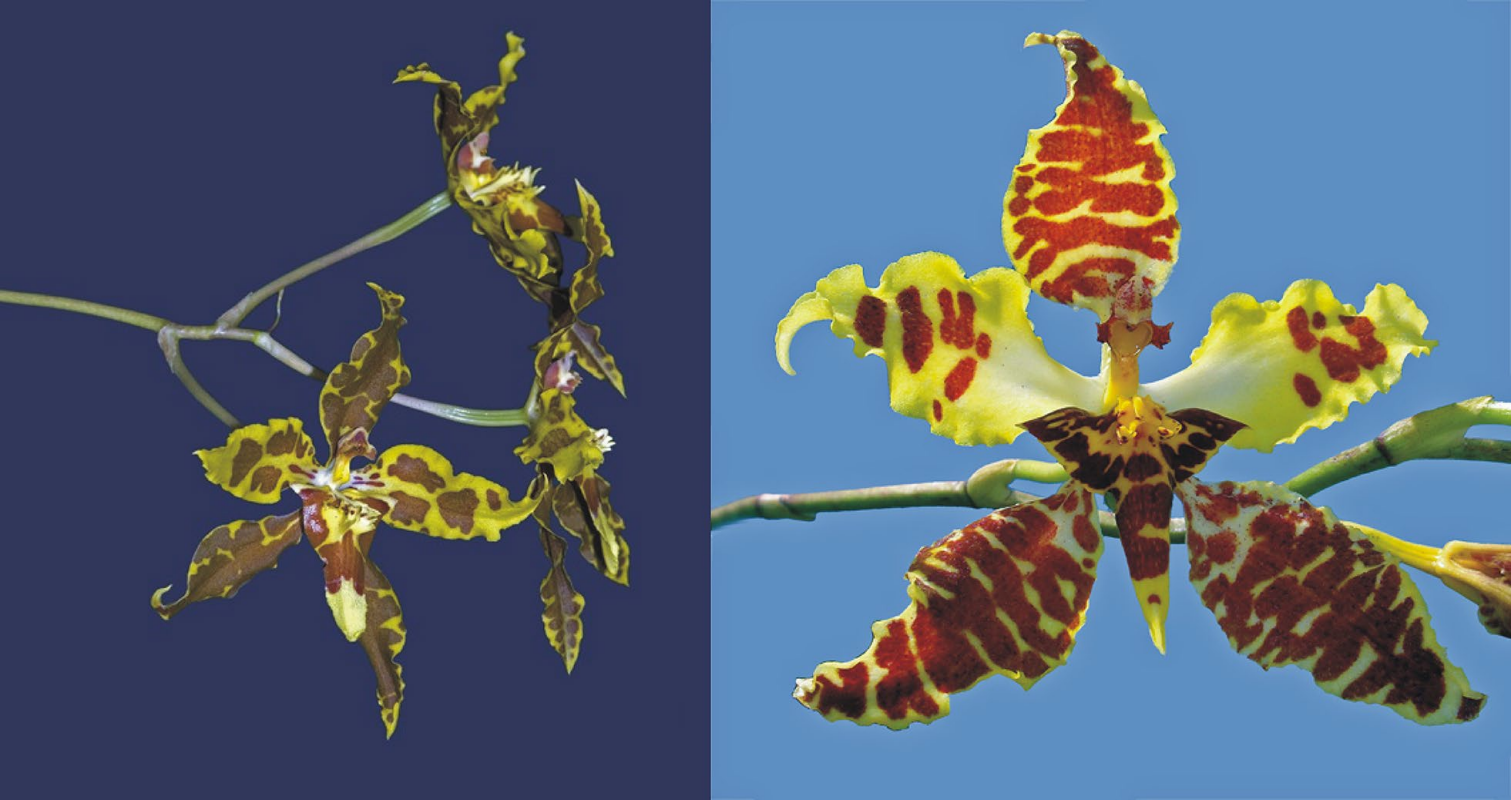
*Cyrtoglossum* flowers. Photos provided by Ecuagenera

There is another question about the potential hybridization between the *Cyrtochilum* and *Odontoglossum* species. Orchids are especially prone to hybridization, partly due to the frequent weakness or even absence of post-zygotic barriers to gene exchange. Instead, many orchids rely heavily on pre-zygotic barriers, notably pollinator preference (e.g. Tremblay et al. 2005; Schiestl and Cozzolino 2008). Orchids, in general, are adapted to various forms of zoogamy and their flowers are accommodated to pollination by various factors, which is one of the reasons for the high degree of variability in the flower, androecium and gynoecium structures. Unfortunately, there are only a few reports concerning this phenomenon in *Cyrtochilum* and *Odontoglossum*. Van der Pijl and Dodson (1966) noted *Bombus hortulanum* and *Centris* bees pollinating *Cyrtochilum macranthum.* Van der Cingel (2001) assumed that the bright-flowered, high-elevation “*C. retusum*” might be hummingbird-pollinated. The flowers of *Odontoglossum s.str.* have also been observed to be pollinated by *Bombus hortulanum* (van der Pijl and Dodson 1966) trying to find reward in the cavity formed by the basal part of the lip and gynostemium. The presence of a hairy lip in some *Odontoglossum* species might suggest pollination conducted by certain callus-collecting insects. In other words, there is a distinct possibility that the species of both *Cyrtochilum* and *Odontoglossum* coul share the same or similar pollination agents, which enable gene flow between plants representing different genera. We have assumed that *Odontoglossum*-like *Cyrtochilum*, i.a. “*C. angustatum*”, “*C. pardinum*”, “*C. ramosissimum*”, could be of hybrid origin or, at least, demonstrate a stronger influence of genetic materials from *Odontoglossum*. It is noteworthy that in 2004 Shaw registered an artificial hybrid between *Odontoglossum* and *Cyrtochilum* named × *Cyrtoglossum* (Fig. 21). The hybrid is characterized by clawed tepals (with petals claws short and wide), and slightly sigmoid gynostemium with winged projections on both sides of stigma. Interestingly, the form of the lip, i.e. oblong obtriangular, with exposed callus reminds *Cyrtochilum macranthos*.

The evolution of sister species is not always combined with parallel evolutionary shifts in pollination syndromes (Cozzolino and Widmer 2005). As a consequence, these closely related species, usually growing in sympatry and having overlapping flowering periods and non-specific pollination are exposed to ample opportunities for inter-specific hybridization (van der Cingel 2001; Jersáková et al. 2006).

It is worthy to mention that the paper published by Stegemann et al. (2012) concerning horizontal gene transfer (HGT), which sheds new light on an incongruity between molecular datasets of various origin. The authors discovered that chloroplast genomes can be readily transferred between relatively closely related species by natural grafting, thus also providing a possible explanation for why chloroplast sequences frequently provide trees that disagree with canonical phylogeny and/or trees constructed with nuclear markers.

It is also possible that portions of genomes can be transferred between even very distantly related lineages via, for example, viral vectors (Won and Renner 2003; Bergthorsson et al. 2004). This process was noticed as a possible cause of erroneous tree topologies by Tyteca and Klein (2008). On the other hand, Tsai et al. (2010) obtained heterogeneous plastid DNA within individuals of two *Phalaenopsis* species—*P. lowii* and *P. gibbosa*. The short DNA fragments in both the *trn*L intron and the *atp*B-*rbc*L intergenic spacer also had indels based on the sequence alignment. One explanation for the different copies of plastid DNA within an individual is the fact that the short form of the plastid DNA might be maintained in the nuclear or mitochondrial genome through horizontal gene transfer (Ellis 1982; Cheung and Scott 1989; Ayliffe and Timmis 1992; Ayliffe et al. 1998). Horizontal transfer can occur in large fragments coming from the organellar genome into the nuclear genome (Yuan et al. 2002; Huang et al. 2005).

Taking into consideration the aforementioned reports, both HGT as well as hybridization might be responsible for the topology of phylogenetic trees and the puzzling position of some species. Reconstruction of evolutionary history in genera strongly affected by reticulation and polyploidization is definitely not an easy task. Importantly, *Cyrtochilum* and *Odontoglossum* species inhabit similar geographical regions and plant communities, i.e. Andean humid montane and premontane forests, often growing together, which facilitates gene/genome transfer. The Andes are a well-known centre of biodiversity, where the process of speciation can take place freely as a result of various geographic conditions (cf. Bates et al. 2008; Richter et al. 2009), and ecological factors. Of course, it could be one of many other explanations for the observed topology of the *Cyrtochilum* phylogenetic tree.

Intraspecific gene evolution cannot always be represented by a bifurcating tree model. Linder and Rieseberg (2004) and Vriesendorp and Bakker (2005) have pointed to the fact that the evolutionary history of many plant groups does not follow divergent evolutionary patterns, and hardly can be unravelled in a tree-building procedure. Rather, it is like a network, which displays a number of reticulate evolutionary events. The family Orchidaceae is not an exception, and poliploidy and hybridization are events, which often result in a reticulated pattern of evolution.

The incompatibility of gene trees does not necessarily constitute evidence for reticulate evolution, as gene phylogenies may conflict with other processes: the gene trees may not be historically accurate due to model misspecification or inappropriate methodology, or due to sampling effects (i.e. insufficient sites to compensate for site saturation or short interior edges); alternatively, the gene phylogenies may be historically correct but differ from the species tree due to the population-genetic effect known as lineage sorting (Maddison 1997; Sang and Zhong 2000; Rosenberg 2002).

To most taxonomists, classification depends on characteristics and we have assumed that to cladists the features are no longer important. A classification should be able to recognize distinctive characteristics which have evolved in a group and if we cannot do that it is in consequence impossible to reflect evolution (Brummitt 2006). Brummitt’s point of view is shared by many other authors (see 150 scientists who signed a letter by Nordal and Stedje 2005), who state that the traditional classification is the optimal tool for cataloguing biodiversity and requires the recognition of paraphyletic taxa.

The dilemma with which every taxonomist has to struggle is fragmentation or lumping of taxonomic units. Both of them can generate various problems. The effect of the fragmentation of taxa is the creation of numerous smaller, but morphologically well-defined genera, whereas integration produces fewer taxa, well-established genetically, but poorly circumscribed morphologically. The borders between taxonomic units are rather a matter of taxonomic philosophy than scientific objectivity. In our opinion, reasonable fragmentation of taxa can be accepted as long as it leads to separation of well-defined entities. We deal with the latter situation in the case of *Cyrtochilum s.l*. We hypothesise that groups of species forming the particular subclades presented above make well-matched genera which evolve in various directions in response to pollinator pressure, which is manifested, e.g. in various spans between lip and gynostemium and the type of the lip calli.

Interestingly, molecular taxonomists attempt to appoint their taxa using morphological characteristics. Unfortunately, they often create ill-defined units and *Cyrtochilum* sensu *latissimo* is an example. Until more data become available concerning the influence of horizontal gene transfer, as well as hybridization and polyploidy on speciation in the *Cyrtochilum* alliance, thus enabling the solution of the problem of incongruity between molecular and morphological datasets, we suggest maintaining a narrower generic concept.

As stated at the beginning of this chapter, Neubig et al. (2012) definition of *Cyrtochilum* does not warrant the proper identification of the genus representatives and can lead to the confusion. We propose to recognize at the generic level smaller, monophyletic and morphologically well-defined taxa, what in our opinion assure stability in taxonomy of this interesting oncidoid group.

### Taxonomic treatment

Key to the genera of *Cyrtochilum*-complex

- 1. Gynostemium and the lower part of the lip form more or less a right angle … 2
- 1*. Gynostemium parallel with the lower part of the lip … 4
- 2. Lip unguiculate, lamina more or less transversely elliptic, callus rather obscure, lateral sepals basally connate … *Siederella*
- 2*. Lip sessile to subsessile, lamina cordate, sagittate to hastate, callus prominent, lateral sepals free to the base or almost to the base … 3
- 3. Lip much smaller than tepals, callus very large, complexed, composed of horns and various digitate projections … *Cyrtochilum*
- 3*. Lip as large as tepals, triangular-cordate in outline, callus large, composed of large mass of tissue divided into 4 or more lobes … *Trigonochilum*
- 4. Lip callus simple, consisting of 2 fleshy, parallel, adjoining torus, diverging in front … *Dasyglossum*
- 4*. Lip callus not as above … 5
- 5. Gynostemium with digitate projections on each sides of the stigma, lip sessile, callus prominent … *Neodryas*
- 5*. Gynostemium with or without very obscure projections, lip long clawed, callus obscure … *Rusbyella*
- *Cyrtochilum* Kunth
- Nov. Gen. Sp. 1: 279. 1816; Generitype: *Cyrtochilum undulatum* Kunth.

Epiphytic or terrestrial plants. Pseudobulbs ovoid, usually round in cross-section, distributed on elongate creeping rhizome, sometimes caespitose, but clusters of pseudobulbs usually distantly remote along the rhizome, with 2–6 foliaceous bracts. Leaves 2–4 per pseudobulb, conduplicate, articulate. Inflorescence flexuose, usually very long, branched, branches with few to many flowers. Flowers resupinate, showy, white, yellow, pink, brown or purple. Floral bracts large, leafy. Tepals free, prominently unguiculate, similar in size and shape or petals much wider. Lip triangular to ovate rarely hastate to panduriform, apex reflexed, callus complex, often digitate, tuberculate or horned. Gynostemium gently sigmoid to erect, elongated, slender, clavate, usually forms right angles with lip. Anther subventral, incumbent, operculate, ellipsoid-ovoid. Pollinia 2, oblong ellipsoid, hard, unequally and deeply cleft, empty inside. Stigma large, elliptic, deeply concave. Rostellum short. Viscidium single, relatively large, elliptic, very thick, concave in the centre of the outer surface. Tegula single, very small, transversely elliptic-obtriangular, obscurely bilobulate at the apex, thin, lamellate. Rostellum remnant with oblique shallowly concave plate at the apex, canaliculate on the upper surface (Fig. 1). Capsule triangular.

Taxonomic notes—Species of the genus *Cyrtochilum*, as treated here, are easily distinguishable from all other genera of the clade by having elongated, flexuose inflorescence, large, showy flowers with clawed, large sepals and petals, a relatively small lip widest at the base, attenuated towards apex, with a composed callus occupying a large portion of the lip lamina, and gynostemium gently sigmoid to erect, slender, usually perpendicular to the lip base, or even deflexed. We assume that a group of *C. angustatum*-group can be of hybrid origin.

- *Dasyglossum* Königer & Schildh.
- Arcula 1: 5 1994; Generitype: *Dasyglossum myanthum* (Lindl.) Königer & Schildh. [≡*Odontoglossum myanthum* Lindl.].

Epiphytic plants. Pseudobulbs approximate, ovoid or elliptic-oblong, compressed, enveloped at the base by papery or foliaceous sheaths. 1–3 leaves, coriaceous or fleshy. Inflorescence usually elongated, erect or arching, few to many flowers, racemose or paniculate. Flowers small. Floral bracts rudimentary. Sepals free, occasionally basally connate, subequal, spreading. Petals usually subequal to the dorsal sepal. Lip entire or 3-lobed, at the base united with the base of the column, lower half of the lip parallel to the column; callus simple, often consisting of 2 fleshy, parallel, adjoining tori, diverging in front, mostly enclosed by the thickened flanks of the gynostemium. Gynostemium rather short, in the upper half gently upcurved or straight, rather robust. Column part ca. 2.5 times longer than anther, fused with the lip in the basal half, firmly winged, wings entire on margins, surrounding lip callus. Anther subdorsal to apical, operculate, ellipsoid, obscurely 2-chambered. Pollinia 2, oblong ellipsoid-ovoid, hard, unequally and deeply cleft. Stigma oblong to transversely elliptic, slightly concave. Rostellum suberect to pendent, rather short, ovate, rounded at the apex. Viscidium single, oblong ellipsoid, very thick, fleshy. Tegula single, as long as viscidium, oblong, thin, lamellate, flat. Rostellum remnant bilobulate at the apex (Fig. 10).

Taxonomic notes—All representatives of the genus are easily separable from other taxa of the *Cyrtochilum* alliance by the presence of a short, massive gynostemium, winged on the ventral surface, and parallel with the lower part of the lip.

The following new combinations are validated below:

- ***Dasyglossum colobium*** (Dalström) Szlach., Kolan. & Chiron, *comb. nov.*
- Basionym: *Cyrtochilum colobium* Dalström *in* Dodson & Luer, Fl. Ecuador, Orchidaceae 87: 51. 2010. Type: Ecuador. Sucumbíos, cloud forest W of La Bonita. 29 Mar 1992. *Dalström & Höijer 1687* (Holotype: SEL).
- ***Dasyglossum ferrugineum*** (Dalström & D. Trujillo) Szlach., Kolan. Chiron, *comb. nov.*
- Basionym: *Cyrtochilum ferrugineum* Dalström & D. Trujillo *in* Dodson & Luer, Fl. Ecuador, Orchidaceae 87: 74. 2010. Type: Ecuador. *Sine loc. hort. Beckendorf sub Dalström 2377* (Holotype: SEL).
- ***Dasyglossum fidicularium*** (Dalström) Szlach., Kolan. & Chiron, *comb. nov.*
- Basionym: *Odontoglossum fidicularium* Dalström, Lindleyana 14(3): 168. 1999. Type: Ecuador. Zamora-Chinchipe. Along road between Yanga and Valladolid. 21 Feb 1982. *Luer* et al*. 7134* (Holotype: SEL, Isotype: K).
- ***Dasyglossum hoeijeri*** (Dalström) Szlach., Kolan. & Chiron, *comb. nov.*
- Basionym: *Odontoglossum hoeijeri* Dalström, Lindleyana 14(3): 171. 1999. Type: Ecuador. Loja. North of Loja, along road to Saraguro. 8 Feb 1993. *Dalström* et al*. 1871* (Holotype: SEL, Isotype: K).
- ***Dasyglossum sphinx*** (Dalström & G.Calat.) Szlach., Kolan. & Chiron, *comb. nov.*
- Basionym *Cyrtochilum sphinx* Dalström & G.Calat., *in* Dodson & Luer, Fl. Ecuador, Orchidaceae 87: 170. 2010. Type: Peru. Cajamarca. Prov. San Ignacio, San José de Lourdes. *Calatayud 746* (Holotype: CUZ).
- ***Dasyglossum verrucosum*** (Dalström) Szlach., Kolan. & Chiron, *comb. nov.*
- Basionym: *Cyrtochilum verrucosum* Dalström *in* Fl. Ecuador 87: 183. 2010. Type: Ecuador. Morona-Santiago. *Ecuagenera sub Whitten 3219* (Holotype: QCA).
- *Neodryas* Rchb.f.
- Bot. Zeit. 10: 835. 1852; Generitype: *Neodryas rhodoneura* Rchb.f.
- *Buesiella* C.Schweinf., Bot. Mus. Leafl., Harvard Univ. 15: 153. 1952; Generitype: *Buesiella pusilla* C.Schweinf.

Plants caespitose. Pseudobulbs cylindrical-ovoid, enclothed basally with 1–3 leafy sheaths, unifoliate. Inflorescence more or less branching, branches with few to many flowers. Flowers medium-sized, campanulate, somewhat laterally compressed. Sepals narrower than petals, both sessile. Lateral sepals variously connate. Lip sessile, entire, oblong-ovate to ligulate-subcordate, geniculate near the middle, callus large, variously lobed. Gynostemium almost erect, stout, parallel to the lip. Column part ca. twice longer than anther, widened in the middle, wings spread, oblong-elliptic, with digitate projections on each side of the stigma. Pollinia 2, obliquely obovoid-ellipsoid, slightly dorsiventrally compressed, hard, unequally and shallowly cleft at the apex. Apical clinandrium forms a narrow collar surrounding the anther base. Stigma rather large, transversely elliptic, deeply concave. Rostellum suberect, short, ligulate. Viscidium single, small, elliptic-ovate, thin. Tegula single, oblong-elliptic, thin, lamellate. Rostellum remnant bilobulate at the middle, slightly concave between acute lobules, canaliculate on the dorsal surface.

Taxonomic notes—We did not identify any morphologically crucial differences between *Neodryas* and *Buesiella*. Therefore, we have combined them together which is also supported by the results of the analyses of the DNA markers. The genus is characterised by the presence of a gynostemium parallel with a sessile lip furnished with a large callus.

- ***Neodryas fredericae*** (Dalström) Szlach., Kolan. & Chiron, *comb. nov.*
- Basionym *Cyrtochilum fredericae* Dalström, *in* Dodson & Luer, Fl. Ecuador, Orchidaceae 87: 86. 2010. Type: Ecuador. Loja. Cajanuma. *Ecuagenera sub Dalström 2478* (Holotype: SEL).
- *Rusbyella* Rolfe *ex* Rusby
- Mem. Torrey Bot. Club 6: 122. 1896; Generitype: *Rusbyella caespitosa* Rolfe *ex* Rusby.

Plants caespitose. Pseudobulbs oblong to cylindrical-ovoid, slightly laterally compressed, enclothed basally with 1–2 leafy bracts, unifoliate. Inflorescence erect, simple or branching. Flowers medium-sized. Sepals and petals subsimilar, narrow. Lateral sepals variously connate. Lip clawed, claw narrow, channelled, furnished at the apex with quadrilobed callus, lamina transversely elliptic to reniform, abruptly reflexed. Gynostemium elongated, slightly swollen and bent back at the apex, parallel with the lower part of the lip. Column part ca. 1.5 times longer than anther, glabrous, with two wing-like projections on both sides of the stigma. Anther subdorsal, incumbent, operculate, dorsiventrally compressed, obovoid-ellipsoid. Connective narrow, thin, forming prominent, apical, roof-like projection in front. Pollinia 2, obliquely obovoid, shallowly cleft at the apex, hard. Apical clinandrium forms a narrow collar-like structure surrounding the anther base. Stigma rather small, oblong elliptic, concave. Rostellum suberect, ligulate, rounded at the apex. Viscidium very small, single, elliptic, thin. Tegula single, linear, thin, lamellate. Rostellum remnant with oblique, apical plate on the inner surface surrounded by obscure fovea.

Taxonomic notes—*Rusbyella* is easily separable from other genera of the *Cyrtochilum* alliance by having a lip with a long, channelled claw, and parallel gynostemium with an apical part and anther bent back.

- *Siederella* Szlach., Mytnik, Górniak & Romowicz
- Biodiv. Res. Cons. 1–2: 5. 2006; Generitype: *Siederella aurea* (Lindl.) Szlach., Mytnik, Górniak & Romowicz [≡*Oncidium aureum* Lindl.]

Plants caespitose. Pseudobulbs ovoid, somewhat laterally compressed, enclothed basally in some leafy sheaths, usually unifoliate. Inflorescence erect, loosely several-flowered. Flowers rather large, showy; sepals and petals subsimilar, sessile. Lateral sepals connate almost to the apex. Lip shortly unguiculate, callus variously developed—almost missing or two parallel ridges, lamina obovate to pandurate. Gynostemium slightly arched, elongated, slender, forming a 30° angle with the lip. Column part ca. 3 times longer than anther, basally joined with the lip, with two projections near the stigma, more or less finger-like. Anther subventral, incumbent, operculate, ellipsoid-obovoid, obscurely 2-chambered. Connective narrow, indistinctly apically elongate. Pollinia 2, obliquely ellipsoid, hard, unequally and deeply cleft, empty inside. Caudiculae sticky, amorphous. Apical clinandrium forms a narrow collar-like structure around the anther base. Stigma large, elliptic, deeply concave. Rostellum shortly conical-digitate in the middle, ligulate, blunt. Viscidium single, rather large, oblong elliptic, very thick. Tegula single, small, linear, thin, and lamellate (Fig. 18).

Taxonomic notes—This genus differs from *Rusbyella* in terms of gynostemium and lip morphology. The lip claw possesses two, parallel elevated calli. The gynostemium forms an acute angle with the lip, it is terete below the stigma, and with small, finger-like projections near the stigma.

### Incertae sedis

**Fig. 22:**
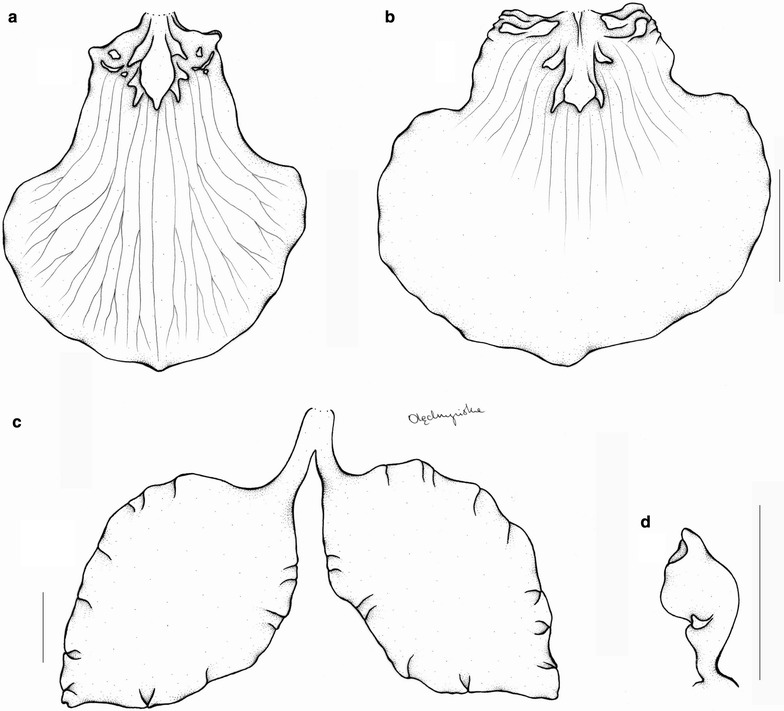
*Cyrtochilum volubile*. **a**, **b** Lip, **c** lateral sepals, **d** gynostemium. Drawn by N. Olędrzyńska. *Scale bar* 5 mm

*Oncidium loxense* Lindl. is similar to *Cyrtochilum* Kunth with which it shares a similar habit, creeping rhizome, long, flexuose, branching inflorescence, large flowers, clawed sepals and petals and gynostemium forming a right angle with the lip. Differs from most species of the genus by having connate lateral sepals, a large, prominently unguiculate lip, with an obscure callus, transversely elliptic or obreniform, concave lip lamina. The lip is somewhat similar to *C. volubile* (Fig. 22) and *C. villenaorum*, but despite these species it is prominently clawed and callus is obscure. Temporarily, we propose to maintain this species in *Siederella*.

- ***Siederella loxense*** (Lindl.) Szlach., Kolan. & Chiron, *comb. nov.*
- Basionym: *Oncidium loxense* Lindl., Paxt. Fl. Gard. 2: 128. 1851. Type (Dalström 2010): Ecuador. *Hartweg s.n.* (Lectotype: K-L, Isolectotype: W).
- *Trigonochilum* Königer & Schildh.
- Arcula 1: 13. 1994; Generitype: *Trigonochilum flexuosum* (H.B.K.) Königer & Schildh. [≡*Cyrtochilum flexuosum* H.B.K.].

Epiphytic plants. Pseudobulbs approximate, ovoid or elliptic-oblong, compressed, enveloped at the base by papery or foliaceous sheaths. 1–3 leaves, coriaceous or fleshy. Inflorescence from the base of pseudobulb, usually very long, twining, frequently with short, zigzag branches. Flowers relatively small. Floral bracts small. Sepals free, subequal, spreading. Petals usually subequal to the dorsal sepal. Lip triangular-cordate in outline, diverging from the gynostemium at 70°–90° with a simple, torous, sometimes verrucose or gibbous callus. Gynostemium elongate, slightly sigmoid to erect, rather slender. Column part 2–4 times longer than anther, with deltoid tabula infrastigmatica, obscurely winged near the stigma, glabrous, wings triangular, entire on margins. Anther subventral, incumbent, operculate, ellipsoid-ovoid, obscurely 2-chambered. Pollinia 2, oblong obovoid, slightly dorsiventrally flattened, hard, unequally and deeply cleft. Stigma elliptic, deeply concave. Rostellum short, conical-digitate in the middle, blunt. Viscidium single, very small, elliptic, rather thick. Tegula single, oblong ovate, thin, lamellate, with roof-like projection above viscidium. Rostellum remnant bilobulate at the middle (Fig. 12).

Taxonomic notes—Considering the flower structure, the genus appears to be similar to *Cyrtochilum*. In both taxa, the gynostemium is more or less perpendicular to the lip base, but unlike *Cyrtochilum*, in *Trigonochilum* the petals and sepals are sessile and the lip is wider than the tepals, lip lamina is triangular-cordate in outline in major part occupied by a massive callus.

Our course of study on the North Andean orchids revealed the existence of an undescribed *Trigonochilum* species from the Colombian Department of Putumayo, as well as the necessity of including into the genus Peruvian, Ecuadorian and Colombian species of *Cyrtochilum*.

**Fig. 23:**
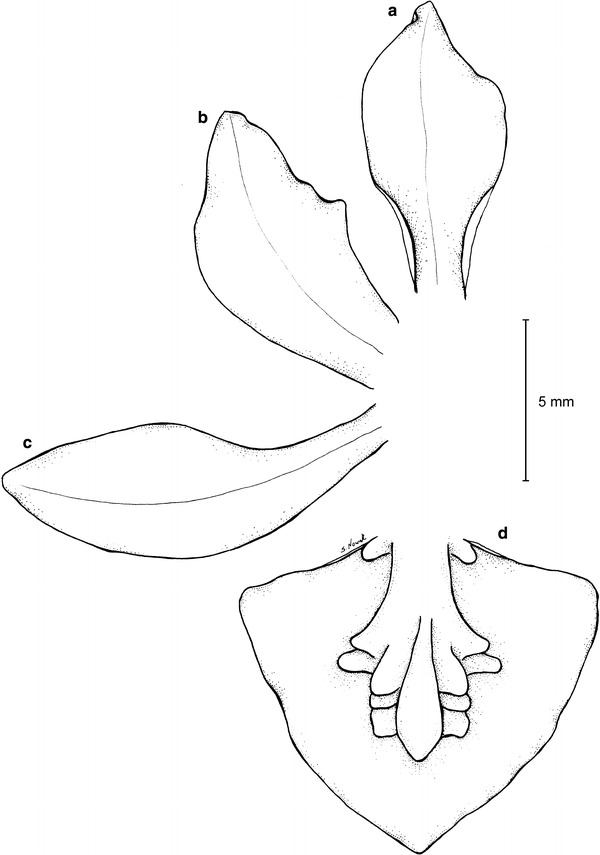
*Trigonochilum koenigerii*—dissected perianth. **a** Dorsal sepal, **b** petal, **c** lateral sepal, **d** lip. Drawn by S. Nowak from the holotype

**Fig. 24:**
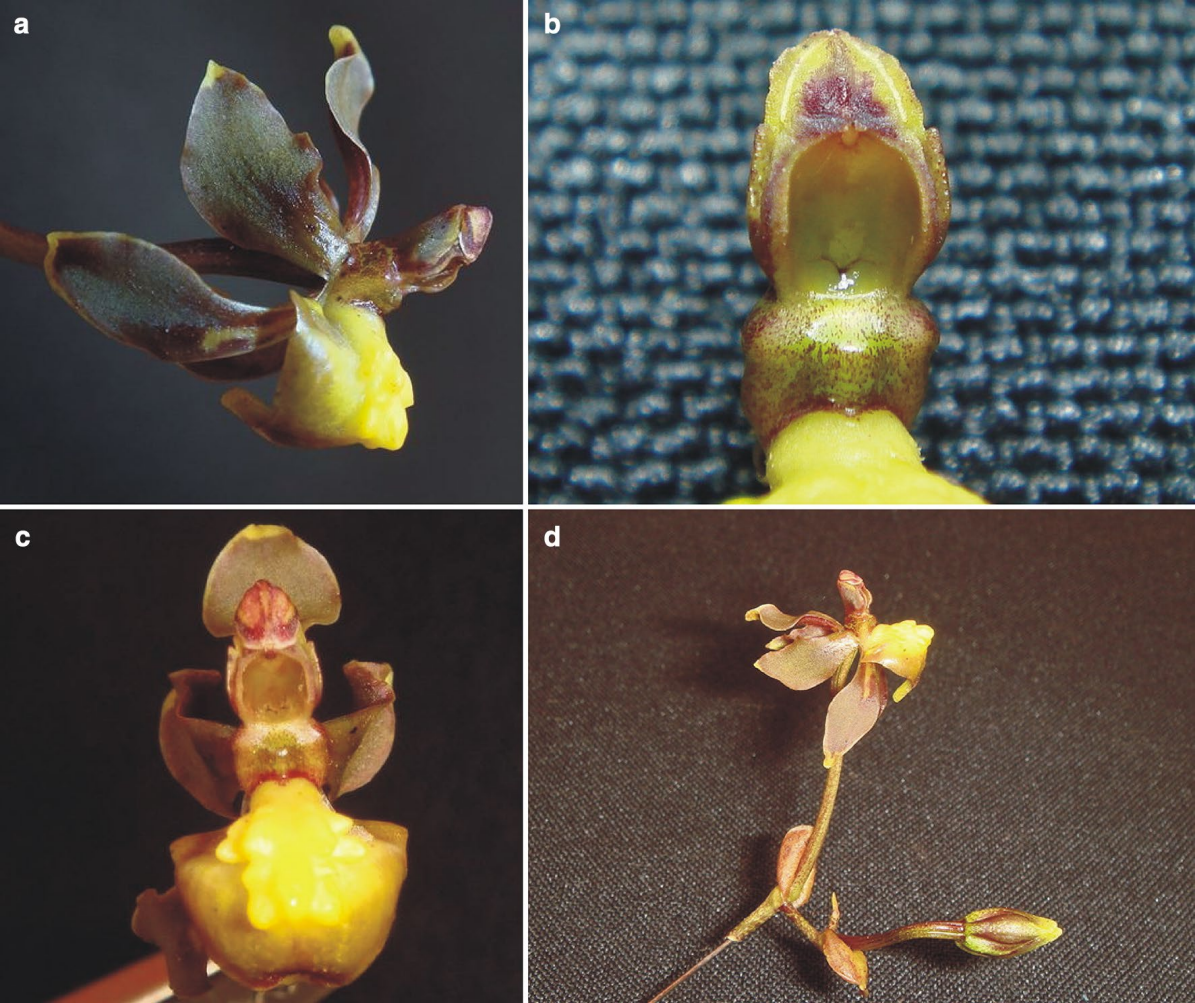
*Trigonochilum koenigerii*. **a** Flower (*side view*), **b** gynostemium, **c** flower (*front view*), **d** fragment of the inflorescence. Photos: R. Medina
- The species is similar to *T. cimiciferum* and *T. midas*, but easily separable from both by the flower color, which is brown or brownish, and complicated lip callus. Additionally, it differs from *T. cimiciferum* by oblong elliptic-obovate, acute petals and large subquadrate stigma, from *T. midas* by acute lip.
- Type: COLOMBIA. Dept. Putumayo. Valle de Sibundoy. Vereda San Pablo Bajo en Km 3 lado izquierdo de la carretera. Cult. *R. Medina 325* (Holotype: MEDEL!).

- ***Trigonochilum corniculatum*** (Dalström) Szlach., Kolan. & Chiron, *comb. nov.*
- Basionym: *Cyrtochilum corniculatum* Dalström *in* Dalström & Perez, Lankesteriana 12(3): 147. 2012. Type: Colombia. Antioquia. Yarumal, Km 87 along road Medellín-Yarumal, Llanos de Cuiba [Cuiva]. 12 Sep 1984. *Dodson* et al*. 15264* (Holotype: RPSC, Isotype: MO).
- ***Trigonochilum midas*** (Dalström) Szlach., Kolan. & Chiron, *comb. nov.*
- Basionym: *Cyrtochilum midas* Dalström, *in* Dodson & Luer, Fl. Ecuador, Orchidaceae 87: 136. 2010. Type: Ecuador. Morona-Santiago. Macas-Guamote. Jan 1989. *Hirtz* et al*. 4061* (Holotype: RPSC).
- Note: Orchid collection of RPSC was transferred to MO.
- ***Trigonochilum russellianum*** (Dalström & Ruíz-Pérez) Szlach., Kolan. & Chiron, *comb. nov.*
- Basionym: *Cyrtochilum russellianum* Dalström & Ruíz-Pérez, Lankesteriana 12(3): 149. 2012. Type: Peru. Ayacucho. La Mar, Aina, Calicanto. *Peruflora sub Dalström 3415* (Holotype: USM).
- ***Trigonochilum sharoniae*** (Dalström) Szlach., Kolan. & Chiron, *comb. nov.*
- Basionym: *Cyrtochilum sharoniae* Dalström, Selbyana 28(2): 106. 2007. Type: Peru. *Sine loc. Peruflora sub Daltröm 2638* (Holotype: SEL).
- ***Trigonochilum tanii*** (Dalström) Szlach., Kolan. & Chiron, *comb. nov.*
- Basionym: *Cyrtochilum tanii* Dalström *in* Dodson & Luer, Fl. Ecuador, Orchidaceae 87: 172. 2010. Type: Ecuador. Manabí, 11 km east of Manta, summit of Cerro Monte Cristi. *Cult. SEL sub Tan 1361* (Holotype: SEL).
- ***Trigonochilum tricornis*** (Dalström & Ruíz-Pérez) Szlach., Kolan. & Chiron, *comb. nov.*
- Basionym: *Cyrtochilum tricornis* Dalström & Ruíz-Pérez, Lankesteriana 12(3): 151. 2012. Type: Peru. Cusco, Quillabamba, Rio Chullapi Reserva. *Valenzuela* et al*. sub*
- *Dalström* et al*. 2699* (Holotype: CUZ).
- ***Trigonochilum koenigerii*** Szlach., Kolan. & Chiron, *sp. nov.* (Figs. 23, 24).

Pseudobulbs about 7 × 4 cm, oblong-cylindrical, apically attenuate, almost rounded in cross section, unifoliate, enclothed basally by 4–6, large, leafy bracts. Leaves up to 38 × 3.5 cm, linear-oblanceolate to linear-oblong, acute, attenuate towards the base. Inflorescence basal, very long, with short, fractiflex, laxly few-flowered branches. Floral bracts 6–8 mm long, ovate-elliptic, obtuse. Pedicellate ovary about 28 mm long, almost straight. Flowers brown or brownish with yellow apices of the petals and yellow lip callus. Sepals free, subequal, spreading. Dorsal sepal about 10 × 5 mm, concave, unguiculate, elliptic-oblanceolate, subacute. Lateral sepals 13 × 6 mm, obliquely spathulate, elliptic-oblanceolate, subobtuse, distinctly clawed. Petals 11 × 0.8 mm, oblong elliptic-obovate, acute, sessile. Lip sessile, 9.7 × 11 mm, entire, convex, widely triangular to ovate-triangular, acute; disc with a fleshy, complicated callus occupying the basal 2/3 and two subglobose thickenings at the base. Gynostemium elongated, slightly sigmoid, slender, forming a right angle with the lip.

Etymology: Dedicated to Willibald Königer, German orchidologist who contributed to our knowledge and understanding of Oncidiinae.

Distribution and habitat: So far, the new species is known exclusively from the Colombian department of Putumayo. It was found growing in disturbed humid montane forest at about 2400 m a.s.l. In cultivation, it flowered in November.

Taxonomic notes: The new species is similar to *T. cimiciferum* (Rchb.f. *ex* Lindl.) Königer from which it differs on the basis of the brown flowers with a yellow lip callus (vs brownish-yellow flowers with brown spots), a complicated lip callus and oblong elliptic-obovate, acute petals. In petal shape, the new species resembles Ecuadorian *T. midas* from which it is easily separable not only in terms of the flower color (almost completely white with a yellow lip callus in *T. midas*) and acute lip (vs lip apiculate), but also the significantly longer ovaries (Table 1).

**Table 1:** Comparative morphology of *Trigonochilum cimiciferum*, *T. koenigerii*, and *T. midas*

Character	*T. cimiciferum*	*T. koenigerii*	*T. midas*
Pseudobulbs	Rounded-ovoid, 5–8 × 1.5–2.5 mm	Oblong-cylindrical, 7 × 4 cm	Ovoid, 4–10 × 2–3 cm
Leaves	Narrowly ovate, 20–40 × 1.5–3 cm	Linear-oblanceolate to linear-oblong, 38 × 3.5 cm wide	Elongate-obovate, acuminate, up to 65 × 2 cm
Pedicellate ovary	10–30 mm	About 28 mm	Up to 20 mm
Flower	Brownish-yellow with brown spots, with yellow lip callus	Brown with yellow lip callus	Almost completely white with whitish to yellow lip callus
Sepals	Spathulate to rotundate, rounded-acute, acute or acuminate	Unguiculate to spathulate, elliptic-oblanceolate, subacute or subobtuse	Spathulate, ovate, acuminate
Petals	Subsessile, obovate-elliptic to narrowly obovate or oblanceolate, acute	Subsessile, oblong elliptic-obovate, acute	Subsessile, ovate, acuminate
Lip	Ovate, ovate-triangular to elliptic-obovate; disc with a fleshy callus in the basal half	Widely triangular to ovate-triangular; disc with a fleshy, complicated callus in the basal 2/3 and two subglobose thickenings at the base	Cordate to truncate; callus a central, longitudinal, fleshy structure, extending from the base to 2/3 of the disc length, terminating in digitate knobs or denticles

### Incertae sedis

The position of “*C. edwardii*” and “*C*. cf. *porrigens*” in the phylogenetic tree is in conflict with results of morphological study, what we discuss above. Partial influence of *Dasyglossum* genetic material can not be excluded in both cases. Because morphological congruence of aforementioned species to other *Trigonochilum* representatives we propose to maintain them temporarily in this genus.

## Additional files

**Additional file 1: Appendix S1.** Specimens of *Cyrtochilum s.l.* and *Odontoglossum* examined during the studies.

**Additional file 2: Appendix S2.** List of the features used in the phenetical study.

**Additional file 3: Appendix S3.** GenBank accession numbers for analysed sequences.
