# Supplementary material for: Taxonomy of Cyrtochilum-alliance (Orchidaceae) in the light of molecular and morphological data
Source: Bot Stud. 2017 Jan 13;58:8. doi: 10.1186/s40529-017-0164-z (PMC5430592; doi:10.1186/s40529-017-0164-z)
Supplement: Supplementary file 2 — Additional file 2: Appendix S2. List of the features used in the phenetical study. [file 40529_2017_164_MOESM2_ESM.doc]

Appendix S1. List of the features used in the phenetical study.

1. Pseudobulbs strongly compressed (0) vs rounded in cross section (1)
2. Plants caespitose (0) vs long-rhizomatous (1)
3. Inflorescence simple (0) vs branching (1)
4. Inflorescence erect/pendulous (0) vs serpentine (1)
5. Flowers campanulate (0) vs flat (1)
6. Tepals subsimilar (0) vs dissimilar (1)
7. Tepals sessile (0) vs clawed (1)
8. Tepals not winged (0) vs winged basally (1)
9. Lip unlobed (0) vs lobed (1)
10. Lip much smaller (0) vs subequal to tepals (1)
11. Lip sessile (0) vs clawed (1)
12. Callus simple (0) vs compoused (1)
13. Claw callus present (0) vs missing (1)
14. Gynostemium and lip form an angle of 30 (0) or not (1)
15. Gynostemium and lip form an angle 90 (0) or not (1)
16. Gynostemium erect (0) or not (1)
17. Gynostemium sigmoid (0) or not (1)
18. Gynostemium free (0) vs connate basally with the lip (1)
19. Gynostemium connate basally along midvein (0) or not (1)
20. Gynostemium slender (0) vs solid (1)
21. Gynostemium winged (0) or not (1)
22. Gynostemium with digitate projections (0) or not (1)
23. Projections below stigma (0) or not (1)
24. Projections above stigma (0) or not (1)
25. Anther terminal (0) vs ventral (1)
26. Viscidium shorter than ½ tegula (0) or longer (1)
27. Tegula with roof-like projection on the inner surface (0) or not (1)
28. Sepals free (0) vs connate basally (1)
29. Tepals strongly undulate (0) or not (1)
